# Metal–Organic Framework Materials for Electrochemical Supercapacitors

**DOI:** 10.1007/s40820-022-00910-9

**Published:** 2022-09-01

**Authors:** Ziwei Cao, Roya Momen, Shusheng Tao, Dengyi Xiong, Zirui Song, Xuhuan Xiao, Wentao Deng, Hongshuai Hou, Sedat Yasar, Sedar Altin, Faith Bulut, Guoqiang Zou, Xiaobo Ji

**Affiliations:** 1 College of Chemistry and Chemical Engineering, Central South University, Changsha, 410083 People’s Republic of China; 2 Department of Chemistry, Faculty of Science, Inonu University, 44280 Battalgazi, Malatya Turkey; 3 Physics Department, Inonu University, 44280 Malatya, Turkey; 4 School of Materials Science and Engineering, Zhengzhou University, Zhengzhou, 450001 People’s Republic of China

**Keywords:** Metal–organic frameworks (MOFs), Electrochemistry, Supercapacitors, Electrode materials

## Abstract

Exploring new materials with high stability and capacity is full of challenges in sustainable energy conversion and storage systems. Metal–organic frameworks (MOFs), as a new type of porous material, show the advantages of large specific surface area, high porosity, low density, and adjustable pore size, exhibiting a broad application prospect in the field of electrocatalytic reactions, batteries, particularly in the field of supercapacitors. This comprehensive review outlines the recent progress in synthetic methods and electrochemical performances of MOF materials, as well as their applications in supercapacitors. Additionally, the superiorities of MOFs-related materials are highlighted, while major challenges or opportunities for future research on them for electrochemical supercapacitors have been discussed and displayed, along with extensive experimental experiences.

## Highlights

- The classification of metal–organic frameworks (MOFs) is summarized: MOFs can be divided into one, two and three dimensions based on the skeletal structure, MOFs can be divided into isoreticular metal organic frameworks, zeolitic imidazolate frameworks, and so on according to the ligand.
- The preparation methods of MOFs are reviewed, which can be divided into traditional one pot method and the emerging preparation methods.
- The application of MOF materials, MOF composite materials, and MOFs-derived materials in supercapacitor is emphasized.

## Graphical abstract

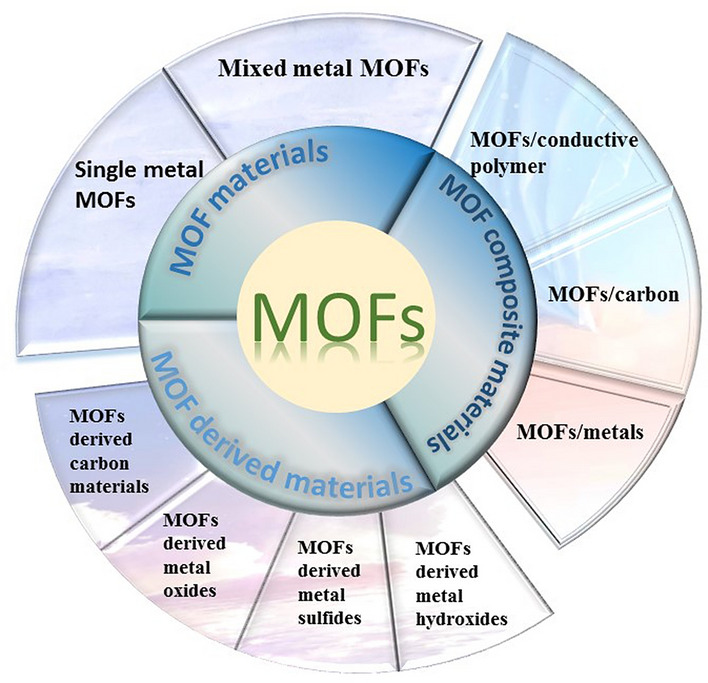

## Introduction

**Fig. 1:**
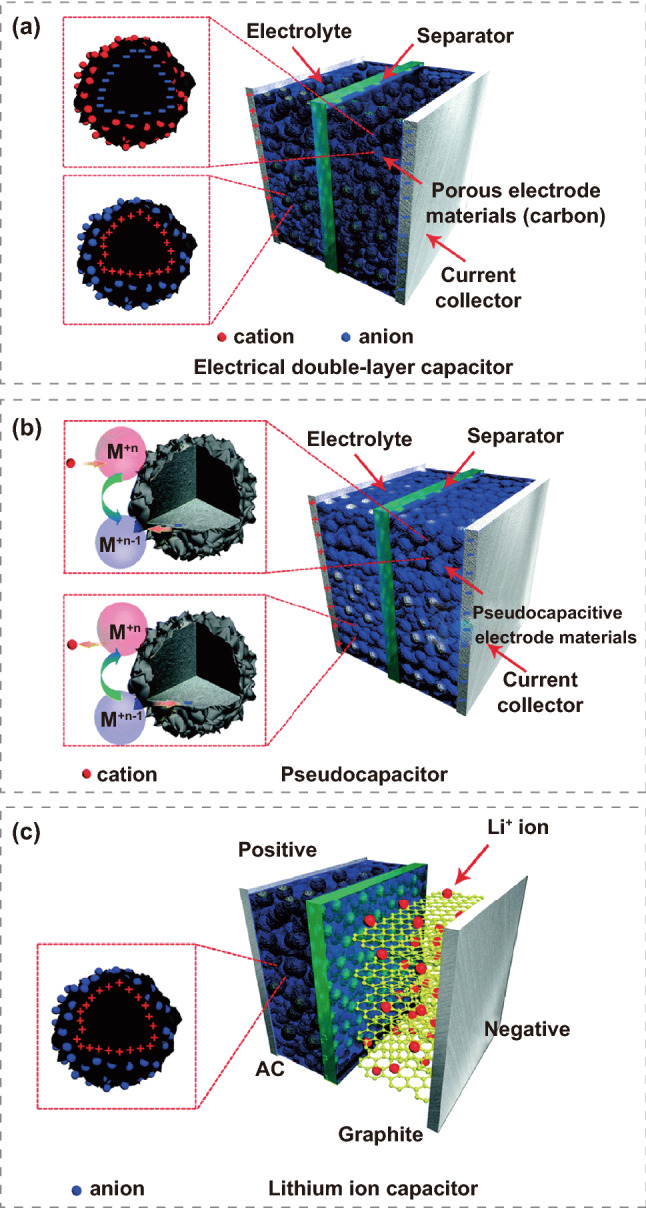
Schematic diagram of **a** an electric double-layer capacitor, **b** a pseudocapacitor, **c** a hybrid-capacitor. Reprinted with permission from Ref. [11]. Copyright 2015 Royal Society of Chemistry

In recent years, the rapid developments in the electronic industries have led to higher requirements for energy storage materials. Environmental pollution caused by the consumption of coal, oil, natural gas, and other non-renewable resources is very serious; thus, developing new green energy storage devices is increasingly fundamental. Supercapacitors, as electrochemical energy storage unit, have broad application prospects in portable devices, hybrid electric vehicles, military industries, national defenses, and other fields due to their superiority of high charging and discharging efficiency, long circular life, and environmental friendliness [1–5]. Supercapacitors can be divided into electric double-layer capacitors (EDLCs) (Fig. 1a), Faraday pseudocapacitors (Fig. 1b), and hybrid capacitors (Fig. 1c), according to the diverse energy storage mechanisms [6–10].

### Highlights

**Fig. 2:**
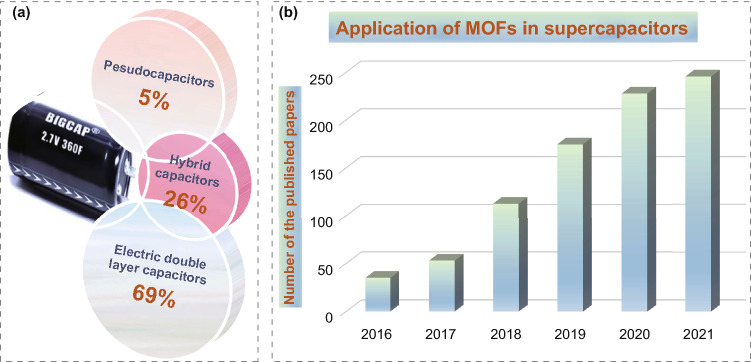
**a** Publications of the ratio of MOFs used in three types of supercapacitors. Data from Web of Science using the keywords “metal–organic framework, double-layer capacitors, faraday pseudocapacitors, and hybrid capacitors.” **b** Publications of MOFs application in supercapacitors. Data from Web of Science using the keywords “metal–organic framework, supercapacitors”

Within EDLCs, the positive and negative ions in electrolyte will be adsorbed on the electrode and electrolyte exterior to generate an electric double-layer structure between the two electrodes during charge/discharge. Complex electrochemical reactions such as chemical bond formation/break will not occur, in contrast to the physical diffusion of positive and negative ions in charge and discharge. Since neither irreversible electrochemical reaction nor phase change of electrode materials occur in the course of charge and discharge, it is only the adsorption and desorption process of electrolyte ions that lead to the relatively long cycle life under high current density. It is generally accepted that the emergence of the electric double layer largely depends on the specific surface area of the electrode/electrolyte interface so that the porous carbon which processes a large specific surface area, also exhibits relatively low cost, satisfactory electronic conductivity, and high utilization rate is seen as an ideal electrode material for EDLCs. Unlike EDLCs store energy by electrostatic adsorption, Faraday pseudocapacitors not only undergo physical diffusion of ions but also carry out chemical reactions during charge and discharge processes, which deliver the higher specific capacitance produced by fast reversible chemical redox reaction than electric double-layer capacitors, leading ten to one-hundred times higher specific capacitance of pseudocapacitance than the electric double layer under the same electrode area. However, due to the insertion/extraction of redox reaction ions on the surface of active materials, the structure will produce defects in continuous expansion and compression until collapse, so the cyclic stability of pseudocapacitors will be slightly lower than that of electric EDLCs. A hybrid capacitor indicates one electrode adopts a traditional battery electrode, which stores and converts energy through an electrochemical reaction, while the other electrode stores energy through an electric double layer, combining the advantages of high power density and good cycle stability of EDLCs, high energy density and high specific capacitance of batteries (Fig. 2a, b). Therefore, the assembled hybrid supercapacitor combines common characteristics and delivers a long cycle life and high power density [12–32].

As the core of a supercapacitor, electrode materials play a key role in its performance [30, 33–36]. To realize the highly efficient energy storage and conversion of supercapacitors, developing novel electrode materials is essential. Metal–organic framework (MOF) materials exhibit a rich framework structure, in which pore size and pore area can be modulated by controlling the proportion of metal salts, organic ligands, solvents, and even some additives. In recent years, MOFs have received much concerns, including their composites and derived materials. Hexagonal Ni-MOF, which largest exposed face (001) crystal plane has the shortest diffusion length beneficial for electron transport and ion diffusion, was synthesized by Pang and coworkers through a one-step solvothermal method [37]. The cross-linked porous network, which grows on the crystal surface, can facilitate the mixing with conductive carbon and thus improve the conductivity and increase the number of active centers. The capacity of 977. 04 F g^−1^ for hexagonal Ni-MOF can be achieved at 0.5 A g^−1^ in the three electrodes and maintain the initial value of 92.34% after 5000 cycles. MOF composites can effectively combine the advantages of MOFs with other functional materials, such as excellent conductivity and unique electrochemical properties, leading to broad application prospects in supercapacitor electrode materials. In another research, Pang and coworkers [38] adopted a one-step coprecipitation means to prepare a novel manganese doped zeolitic imidazolate frameworks-67 (ZIF-67) with a larger size than other ZIF-67 synthesized by the same procedure due to the introduction of manganese ions with a radius larger than that of cobalt ions. Subsequently, this will facilitate contact with the electrolyte and promote ion dispersion and electron transfer. Moreover, the conductivity of the prepared samples is also greatly improved because the mixed metal composition produces the coupling effect of many different metals. When used in supercapacitor electrochemical testing, the capacity of 926.25 F g^−1^ can be achieved at 0.5 A g^−1^ for Co/Mn-ZIF material, as well 64.1% of their initial first cycle was still retained at 10 A g^−1^ even after 1500 circulation. MOFs-derived materials can inherit the structure of precursors and supply a new approach for preparing high-performance electrode materials in supercapacitors. One-dimensional carbon nanorods, which can be transformed into graphene nanoribbons in the subsequent process, were prepared by a thermal transformation without catalyst using rod MOF as a template by the Xu group [39]. The capacity of 198 F g^−1^ can be achieved at 50 mA g^−1^ for it, which showed the best specific capacitance values of carbon and graphene materials reported so far, confirming the availability of these nanoribbons in practical applications. Obviously, it can be seen that the preparation of the required electrode material by using MOFs as a template is simple and efficient.

Some reviews on the application of MOFs in electrochemistry fields have been published in recent years [40–46], but still, there are few introduction to MOF materials [47–49]. Based on this, the types and synthesis strategies of MOFs are analyzed and discussed in this paper. Meanwhile, the application of MOFs mainly focuses on the battery system; actually, the characteristics of large specific surface area and uniform pores mean that it is more suitable to be applied to supercapacitors as electrode material. Thus, we will review the research progress of MOFs in supercapacitors from three aspects mentioned above.

## Classification and Synthesis of MOF Materials

### Classification of MOF Materials

As a new functional material, MOF materials have been widespread applied and investigated recently, resulting in the rapid development of their design and synthesis. Now tens of thousands of MOF materials with different compositions and structures have been synthesized, which greatly increases the degree of difficulty for analyzing. Moreover, the types of MOF materials needed in various fields are different; in order to use them more quickly and efficiently, researchers have reasonably classified them according to different methods. Generally, MOFs can be fell into one-dimensional (1D), two-dimensional (2D), and three-dimensional (3D) skeleton according to the different compositions of their complex skeleton space. Yu et al. [50] used polydopamine (PDA) to control synthesizing 1D MOFs by a facile contra-diffusion synthetic strategy and subsequently extended this strategy to synthesize other one-dimensional MOF superstructures. 2D MOFs, processing abundant active sites and highly ordered pore structure, are widely used in many fields, especially in supercapacitors. Bao et al. [51] reported a high-performance electrode based on 2D MOFs derived from conducting hexaaminobenzene (HAB). HAB MOF particles with submillimeter thickness delivered a high areal capacity of 20 F cm^−2^. There are many kinds of 3D skeleton products, and the structure is also the most complex, including cubic type, diamond type, molecular sieve type, and other structural types [52]. Although this classification method succinctly summarizes the structural characteristics of MOF materials, which is convenient for MOFs with different skeletons to shine in various fields, only skeleton information cannot distinguish the composition of central ions and organic ligands. The most frequently mentioned method is classified according to the type of ligand, not only solving the above shortcomings but also clarifying the characteristics of different MOFs series. It can be divided into isoreticular metal organic frameworks (IRMOFs) according to various ligands, which are microporous crystal materials self-assembled with [Zn_4_O]^6+^ inorganic groups and a series of aromatic carboxylic acid ligands. IRMOF-1 (also known as MOF-5) is a porous material composed of divalent zinc ions and organic ligand p-benzoic acid (PTA) through ligand action in a solvent, which is the most widely used member of this family [53]. Researchers have developed various preparation methods to synthesize MOF-5 materials quickly and efficiently. Cheng et al. [47] synthesized MOF-5 with a penetrating structure by dissolving zinc nitrate hexahydrate and p-phthalic acid (PTA) in N,N-dimethylformamide (DMF) via the solvothermal method (110 °C) in the presence of melamine and found that pH value would affect the synthetic crystal structure. Zeolitic imidazolate frameworks (ZIFs) with zeolite structure are synthesized by the reaction of Zn ions or Co ions with imidazole ligands [54]. ZIF-8 is a polyhedral crystalline cage structure compound synthesized by self-assembly of divalent zinc ions and 2-methylimidazole ligands under mild synthesis conditions compared with other ZIFs [55]. Coordination pillared layer (CPL) series with gate opening phenomenon are composed of six coordinated metal elements coordinated with ligands such as 2,2'-bipyridine or phenol. Gate opening phenomenon means that the crystal structure skeleton of the material will expand significantly at a critical adsorption point and result in great changes in pore structure and sudden changes in the ability to adsorb guest molecules when the material is adsorbing guest molecules. Materials of institute lavoisier (MIL) series take transition metal as the central ion, which biggest feature is the material structure that will change between large and narrow pores under the stimulation of external factors called breathing phenomenon [56]. MIL-100 and MIL-101 are widely studied in which the fronter is centered on ferric ions, and MIL-101 is a cubic structure centered on trivalent chromium ions. Porous coordination network (PCN) series contain several cubic octahedral nanopore cages with enormous potential in the realm of gas storage [57]. University of Oslo (UIO) series have high specific surface area and good chemical and thermal stability, showing certain potential in water environment extraction. UiO-66 is composed of Zr_6_O_4_(OH)_4_ and ligand terephthalate, and the inner surface will form a triangular structure with a large Langmuir surface area, so it is very stable even in a humid environment [58]. The above methods are too cumbersome and not conducive to induction, so we divided them into single metal MOFs and multi-metal MOFs according to the number of central metal ions and introduced their application in the field of supercapacitors.

### Synthesis of MOF Materials

**Fig. 3:**
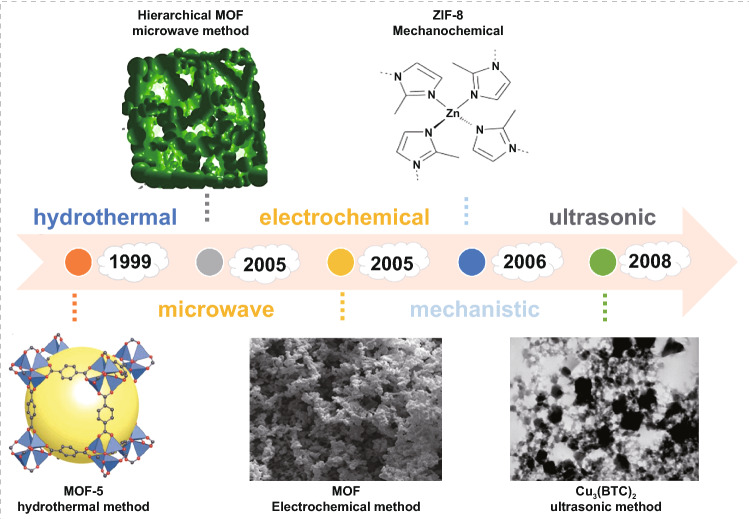
Timeline of the traditional one-pot synthesis. Reprinted with permission from Ref. [63, 68, 70–72]. Copyright @1999 Macmillan Magazines Ltd. @2006 Royal Society of Chemistry, @ 2008 Elsevier, @ 2020 American Chemical Society, @ 2015 Nature Publishing Group

MOF materials with various structures and functional groups can be synthesized via reasonably selecting inorganic metal centers and organic ligands. Currently, MOF materials can be prepared in a variety of ways, which can be summarized as 'typical one-pot synthesis' and emerging preparation methods. Typical preparation methods, including hydrothermal [59], microwave-assisted [60], electrochemical, mechanistic [61], and ultrasonic chemical [62], which is obviously economically and environmentally friendly to obtain complex molecules directly from relatively simple and readily available materials without the separation of intermediates, but cannot effectively predetermine the structure and properties of the target product (Fig. 3). The hydrothermal method is a reaction carried out in an airtight system with high temperature and high pressure, solving the problem that some reactants are insoluble at room temperature with simple operation equipment and low energy consumption. However, the synthesis time is long and difficult to control the crystal morphology. Solvent extraction and crystal formation are realized according to the principle that the increase in solution viscosity leads to the strengthening of diffusion. Yaghi group [63] used this method to synthesize MOF-5 from p-benzoic acid (PTA) in early 1999. Another typical example is the IRMOF series materials synthesized by Eddaoudi et al. [64], which mixed zinc nitrate hydrate with 12 different organic ligands with N,N-Diethylformamide as solvent. Microwave synthesis is the most widely used method in organic chemistry, which can improve the chemical reaction rate since the solvent can rise rapidly in a very short time under the action of microwave, causing the synthesis of MOFs by microwave irradiation that can greatly save the reaction time, sometimes even in tens of seconds [65]. In 2005, the microwave method was firstly used to synthesize MIL-100 by Jhung et al. [66]. The reaction mixture was placed in sealed polytetrafluoroethylene with high-pressure steam in a microwave oven at 220 °C with hydrofluoric acid. The synthesis time of the MIL-100 framework was shortened from 96 to 4 h, and it is worth noting that although the synthesis time was reduced by 24 times, the yield of the product remained unchanged. Two years later, they further reported the successful synthesis of spherical MIL-101 via the same method [67]. In the electrochemical synthesis method, the metal ions are provided by dissolving the anode rather than introducing from the solution of the corresponding salt, which can reduce the number of anions in the reaction process and is conducive to mass production of MOFs. Pastre et al. [68] first proposed the electrochemical synthesis of MOFs in a patent in 2005. Cu-MOF was successfully prepared by anodic synthesis using copper plates with a thickness of 5 mm in a pool of methanol containing 1,3,5-Benzenetricarboxylic acid (H_3_BTC) as the anode and cathode simultaneously. Applying voltage of 12–19 V moreover 150 min, a green–blue precipitate was deposited. More and more MOF materials have been synthesized using electrochemical synthesis, which is maturing and improving. Mechanical stirring is a solvent-free method, which is usually used when a large number of materials are required because this method can reduce not only the environmental pollution caused by solvent volatilization but also saving costs. This method was primarily used in 2006 by Pichon et al. [69], who claimed that a [Cu(INA)_2_]_*n*_ was obtained by ball milling with isonicotinic acid and copper acetate as raw materials under solvent-free conditions for 15 min. Ultrasonic synthesis refers to the use of ultrasonic energy to control the crystallization process of chemical synthesis, which can quickly synthesize uniform particle size nano-MOF materials. However, the reaction is not easy to control and the occurrence of side reactions leads to more impurities, which is not conducive to material performance analysis, but the price is lower and easier to achieve in industrial production. In 2008, Qiu et al. [70] from Anhui University used this method to synthesize Zn_3_(BTC)_2_ for the first time. After zinc acetate and 1,3,5-Benzenetricarboxylic acid (H_3_BTC) were dissolved in an aqueous ethanol solution for 5 min by ultrasonic, a yield of 75.3% MOFs was obtained. In the subsequent progress, some researchers prepared MOF-5 by ultrasonic method, and the corresponding synthesis time was shorter than the microwave heating method.

**Fig. 4:**
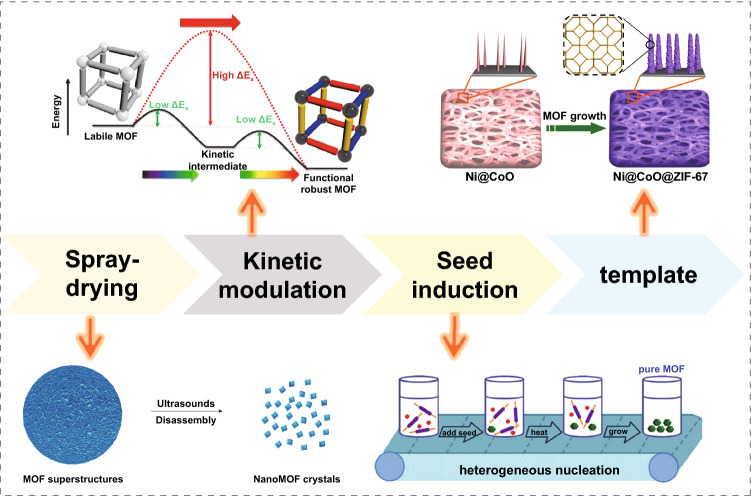
Diagram of emerging preparation methods. Reprinted with permission from Ref. [73–76]. Copyright @2013 Nature Publishing Group, @ 2017 American Chemical Society, @ 2016 American Chemical Society, @ 2018 Springer Science Business Media

Emerging preparation methods such as spray-drying strategy, kinetic modulation, seed induction, and template synthesis are developed to regulate the reaction process and achieve controlled synthesis of MOF materials (Fig. 4). The spray-drying strategy was first proposed by Daniel et al. [73] in 2013 for the synthesis and self-assembly of nanoscale MOFs, which can produce a large number of hollow and spherical sub-5 μm MOF superstructures by local crystallization of nano-MOFs on the surface of atomized droplets of MOF precursor solution after heating. Importantly, this strategy proved to apply to a wide range of MOFs. In 2016, Bosch et al. [74] proposed the concept of dynamically modulated crystal growth to adjust the crystallization, pore size, shape, and accurately control the stability of MOFs by changing reaction conditions such as solution pH or temperature to prepare novel and controllable MOF materials. Seed-induced synthesis introduces the seed of the target product into the reaction system, effectively improving the purity of the MOFs crystalline phase and directly preparing high-purity MOF materials. Xu et al. [75] synthesized single-phase Zr MOF by this method. They fixed other reaction parameters except for the introduction of seed because the nucleation stage was skipped, generating mixed nuclei, and obtaining phase-pure MOF. The template method is often used to prepare porous MOFs. Cai et al. [76] used the nickel-based CoO nanowire array as the template to provide the skeleton for the growth of Co^2+^ and ZIF-67 and obtained the composite array Ni@CoO@ZIF-67 with a uniform rod structure. Duan et al. [77] prepared various HP MOFs with multimodal layered porous structure and good thermal stability at room temperature with a surfactant as a template and zinc oxide as a promoter. Furthermore, the synthesis time is greatly shortened to 11 min.

## Application of MOFs in Supercapacitors

MOFs usually can be divided into three categories when they used as electrode. The first type is to use the MOF material itself as the electrode material, not blended with other materials, which is simple to prepare; however, the conductivity is still poor, resulting in a low specific capacity. The second type is to synthesize the electrode material with MOF and other materials, which conductivity and stability are better than those of MOF electrode materials due to the introduction of new doping components. The third type is to use MOFs derived from other electrode materials containing carbon materials, metal oxides, metal sulfides, or metal hydroxides.

### MOF Materials as Electrode

#### Single Metal MOFs

**Fig. 5:**
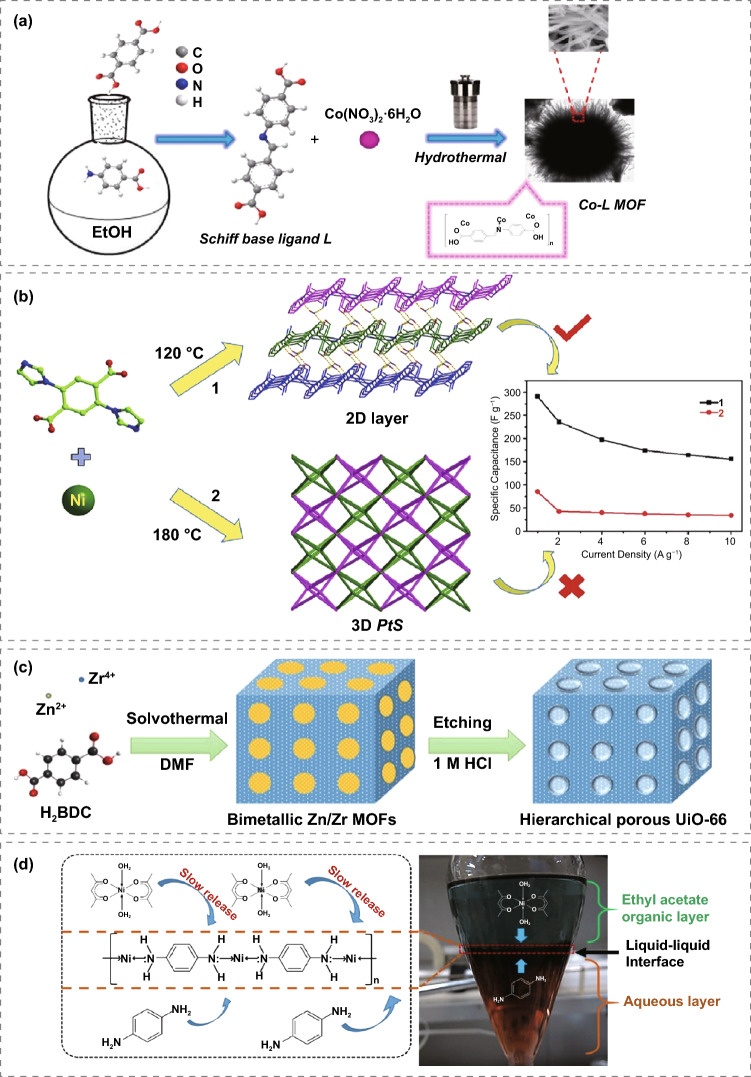
**a** The synthesis diagram of Co-L MOF. Reprinted with permission from Ref. [82]. Copyright 2019 Elsevier B.V. **b** Influence of temperature on synthesis of two Ni-MOFs. Reprinted with permission from Ref. [86]. Copyright 2019 Elsevier Inc. **c** Schematic diagram of preparation process of HP-UiO-66. Reprinted with permission from Ref. [92].Copyright 2017 American Chemical Society **d** Schematic diagram of liquid–liquid interface reaction of Ni-pPDA MOF at room temperature. Reprinted with permission from Ref. [94]. Copyright 2018 Elsevier B.V

Cobalt-based MOFs are the most frequently used transition metal MOFs, playing a significant role in electrochemical field. In 1995, Yagh et al. [78] reported a coordination compound with a 2D structure in nature, with transition metal Co as the center and pyromellitic acid as the organic ligand, and put forward the concept of MOFs for the first time. Moreover, in 2012, Lee et al. [79] primarily reported that Co-MOF was directly used as supercapacitor material, whose specific capacitance of 206.76 F g^−1^ can be achieved at 0.6 A g^−1^. After 1000 cycles, the specific capacitance loss was only 1.5%. Next year they used three dicarboxylic acids with different molecular lengths as organic linkers to regulate the pore size and specific surface area of cobalt-based MOFs [80]. As seen from N_2_ adsorption and desorption tests, Co-BDC, Co-NDC, and Co-BPDC exhibited the pore size (specific surface area) of 2.58 nm (9.09 m^2^ g^−1^), 13.95 nm (20.29 m^2^ g^−1^), and 78.96 nm (138.35 m^2^ g^−1^), respectively. The specific capacitances of 131.8, 147.3, and 179.2 F g^−1^ were achieved under the scanning speed of 10 mV s^−1^, respectively. It is believed that the longer connector leads to the larger pore and the specific surface area, producing better supercapacitor performance. Co-BDPC: a 3D surface structure composed of continuously interconnected lobular microstructure entities, has a large cavity, which provides the free path for charge transfer, but collapses after 1000 cycles. This leads to a large loss of charge storage capacity of it, so the specific capacitance of the supercapacitor constructed by Co-BPDC can only be retained at 84.6% after 1000 cycles of charge/discharge, proving that the specific surface area and pore size have an important influence on the improvement of the capacitance performance of the supercapacitor constructed by MOFs. In order to obtain high-performance supercapacitors, many scholars have invested efforts to find other ideal electrode materials. A new type of Co-MOF with columnar layered structure was synthesized by Abazar et al. [81] in 2019, showing a capacitance of 325 F g^−1^ at 5 A g^−1^, high energy density (50.30 Wh kg^−1^) and long cycle life (the capacitance retention rate is 90.70% after 6000 cycles) when it was used as an electrode on asymmetric supercapacitor. Cobalt-based organometallic frameworks (named Co-L-180) with sea urchin-like structures were synthesized by Zhou group [82] using the hydrothermal method at 180 °C (Fig. 5a), and studying their applications as supercapacitor electrodes. The results showed that the specific capacitance of 404 F g^−1^ for Co-L-180 can be achieved at a current density of 1 A g^−1^ and exhibited excellent cycle stability. The hybrid supercapacitor, using Co-L-180 as a positive electrode and activated carbon as a negative electrode, has an energy density of 21.5 W kg^−1^ at a power density of 684 W kg^−1^.

Nickel-based materials are also very important electrochemical energy storage materials [83]. Kang et al. [84] prepared Ni_2_(BTC)_3_ by solvothermal reaction of nickel chloride and pyromellitic acid (H_3_BTC). The supercapacitor based on the Ni-MOF showed excellent pseudocapacitance performance in KOH electrolyte; when the current density is 1 A g^−1^, the specific capacitance can reach 726 F g^−1^. In addition, it displayed good electrochemical stability, and the capacity retention ratio can still keep 94.6% after 1000 cycles. After 5000 cycles, the capacitance decreased only 65% of the initial capacitance. The main reason for the decrease in capacitance maybe was the reduplicative insertion and removal of OH^−^ at the interface during continuous cycle, which leads to the collapse of the Ni-MOF structure and hinders the electrolyte wetting electrode. Qu et al. [85] reported novel Ni-based columnar MOFs (Ni-DMOF-ADC/TM/NDC) with different kinetic stabilities that were synthesized and applied to supercapacitors. The capacitance of 438 F g^−1^ for Ni-DMOF-ADC can be achieved at 20 A g^−1^. The capacitance retention rate is more than 98% after 16,000 cycles, maintaining excellent cycle stability at 10 A g^−1^. However, Ni-DMOF-TM and Ni-DMOF-NDC can only maintain 60% and 50% of the capacity. Its excellent electrochemical performance is attributed to the transform of DMOFs into highly functional nickel hydroxide, which inherits the high stability of DMOF-ADC and remains intact during the charge/discharge process. Ni-based MOF's structural stability greatly influences its electrochemical properties, which can be effectively improved by synthesizing MOFs with different morphologies. The influence of temperature on the preparation of two kinds of Ni-MOFs in supercapacitors with excellent capability was explored by Deng group [86]. Two new MOFs, Ni-MOF_1_ and Ni-MOF_2_, were synthesized at 120 and 180 °C, respectively, showing that different reaction temperatures can trigger different reaction processes to obtain varied structures (Fig. 5b). Ni-MOF_1_ is a 2D layered structure, and Ni-MOF_2_ has a 3D framework; the electrochemical property of Ni-MOF_1_ is superior to that of Ni-MOF_2_, which may be attributed to the fact that the 2D layered structure is favorable for electron transfer. This result was also mentioned in the work of Yang group [87], who synthesized a nickel-based MOF layered with NiCl_2_·6H_2_O and terephthalic acid (PTA). As electrode material for supercapacitors, the Ni-MOF delivered large specific capacitance of 1127 F g^−1^ at 0.5 A g^−1^, and outstanding cycle stability (more than 90% performance can be maintained after 3000 cycles). Additionally, they expound that this outstanding electrochemical property is attributed to nickel-based MOF's inherent characteristics, layered structure, and highly exposed and uniformly dispersed active sites.

The Ni-MOF with accordion-like morphology was synthesized by Yan et al. [88] through ultrasonic method. It has multilayer thin sheets compared with the non-ultrasonic bulk Ni-MOF, which means there are thousands of nano-channels in the layered structure, greatly improving the diffusion of ions and electrolytes. The specific capacitance of 823 F g^−1^ for accordion-like Ni-MOF can be achieved at 7.0 A g^−1^ and the capacitance retention rate of 96.5% after 5000 cycles is achieved at 1.4 A g^−1^). High conductivity Ni_3_(HITP)_2_ grown on a conductive substrate, which directly served as electrode material (without adding active carbon material) for the first time, was prepared by Sheberla [10]. The area-specific capacitance of the Ni_3_(HITP)_2_-based supercapacitor is about 18 μF cm^−2^, showing a higher area-specific capacity than most carbon-based electrode materials. Simultaneously, after 10,000 cycles of testing, the capacity retention rate is still as high as 90%. The organic framework materials of isonicotinic acid and nickel nitrate were prepared by the solvothermal method as electrode materials for supercapacitors in Liao's report [89]. The effects of solvothermal time, temperature and molar ratio of isonicotinic acid to nickel nitrate on electrochemical property were studied. The results showed that the maximum capacitance of Ni/ISO_4_ is 634 and 457 F g^−1^ at 5 and 10 mV s^−1^, respectively. Even at the scanning rate of 50 mV s^−1^, the specific capacitance of the composite electrode decreases only 16% after 2000 cycles.

Cobalt and nickel are important raw materials for the electrode of supercapacitors, but their reserves are limited and expensive. Researchers are trying to apply Zr-MOFs, Fe-MOFs, and others to prepare supercapacitors. Tan et al. [90] synthesized Zr MOFs (UIO-66) with different specific surface areas by zirconium chloride and hydroquinone (H_2_BDC) at different temperatures (50, 70, 90, and 110 °C) and applied them to electrochemical energy storage. When the scanning speed is 5 mV s^−1^, the specific capacitance of 1144 F g^−1^ for Zr-MOF_1_ is much higher than other Zr-MOFs. The internal resistance (*R*_s_) of them is 0.42, 0.53, 0.60, and 0.62 Ω, respectively, showing that the Zr-MOF_1_ electrode synthesized at 50 °C has the lowest internal resistance. The long cycle test shows that the specific capacitance of 654 F g^−1^ for Zr-MOF_1_ can be maintained after 2000 charging and discharging cycles. The reason might be due to the pore size of UIO-66 changes with the temperature change, and the corresponding aperture of Zr-MOF_1_ can shorten the charge transfer path and accelerate the charge/discharge rate. For another, the larger specific surface area increases the contact surface of electrolyte and active substance, which is conducive to improving electrochemical performance. Choi et al. [91] selected different metal salts and organic ligands to synthesize 23 kinds of crystalline N-MOFs with different pore sizes and shapes. Several members of those series show high capacitance, especially Zr-MOF, which has the highest area-specific capacitance of 5.09 mF cm^−2^, and the volume-specific capacitance of 0.64 F cm^−3^, and mass-specific capacitance of 3726 F g^−1^, respectively. The specific capacitance is about 6 times of those made of reference commercial activated carbon materials. After 10,000 cycles, the specific capacitance has little loss so it is believed that MOF's stable channel structure provides a large channel area for ion transmission, effectively shortening the charge transmission path. More importantly, the high exposure and even dispersed active sites improve the performance of the supercapacitor. Layered porous Zr-MOF (HP-UIO-66) was successfully prepared [92] by removing Zn-MOF from bimetallic Zn/Zr-MOF (Fig. 5c). Due to the large specific surface area, pore-volume, and abundant surface defects of the layered porous structure, HP-UIO-66 as the electrode material of supercapacitor, exhibits a specific capacitance of 849 F g^−1^ at 0.2 A g^−1^, which is 8.36 times higher than 101.5 F g^−1^ of ordinary UIO-66. When the power density is 240 W kg^−1^, the energy density is Wh kg^−1^. Fransaer et al. [93] synthesized three kinds of Fe MOFs named MIL-100 (Fe), MIL-88 (Fe), and MIL-53 (Fe), which used different neutral aqueous solutions as electrolytes to study the effects of pore size, wall thickness, and electrolyte cation on the electrochemical performance. Kannangara et al. [94] synthesized Ni-pPDA, and Mn-pPDA layered MOF electrodes by liquid–liquid interfacial reaction with p-phenylenediamine (pPDA) as an organic ligand (Fig. 5d) and combined them as the positive electrode and negative graphite carbon (GC) electrode to prepare composite supercapacitor. The performances of the supercapacitors using Ni-pPDA//GC and Mn-pPDA//GC are 184.7 and 109.3 F g^−1^, respectively. In particular, Ni-pPDA supercapacitors deliver higher energy and power density after 5000 cycles; the capacitance retention of Ni-pPDA and Mn-pPDA MOFs are 80% and 97%, respectively. The first reason why p-phenylenediamine was chosen as the ligand material is that the lone pair electrons provided by the amine group in pPDA can easily combine with the metal group to form a strong coordination bond. Secondly, pPDA has the following advantages π-conjugated electron cloud that can improve the conductivity. Thirdly, the liquid–liquid interface reaction between pPDA and metal acetylacetone is very easy. Shinde et al. [95] inserted clean and well-foamed nickel substrates into polytetrafluoroethylene (PTFE) liners with reactive solutions and then prepared two-dimensional layered Mn-MOF nanosheets at different reaction temperatures, which exhibited good electrochemical performance in 2 M KOH electrolyte as the electrode for supercapacitor. At 1 A g^−1^, the maximum specific capacity (area capacitance) is 567.5 mAh g^−1^ (10.25 F cm^−2^), and the capacity retention rate is 92.3% after 5000 cycles. The specific capacitance, volume specific capacitance and energy density of the hybrid supercapacitor are 211.37 F g^−1^, 3.32 F cm^−3^, respectively. The intrinsic characteristics of Mn-MOF such as layered structure, open space for effective electrolyte access, and short ion diffusion paths may be the reasons for its excellent electrochemical performance. Rare earth metal elements have unique redox characteristics, leading to their oxides widely used in the design of capacitors. In recent years, researchers have also tried to directly apply rare earth metal MOFs to the development of supercapacitors. Eu-MOF with fumaric acid and oxalic acid as ligands was synthesized by Dezfuli et al. [96] using hydrothermal method. The specific capacitance of 468 F g^−1^ can be achieved at 1 A g^−1^ for Eu-MOF and 260 F g^−1^ at 16 A g^−1^. After 4000 cycles, it still has a capacitance retention of 95.2%. Subsequently, Jafari et al. [97] synthesized Tb-MOF with better capacitance performance by the same method. The capacitance is 510 F g^−1^ at a current density of 1 A g^−1^ and after 4000 cycles still having 91.9% capacitance retention even showing a higher capacitance at 16 A g^−1^.

These results show that the development and application of MOFs in the SCs field can be promoted by improving the structural stability of MOFs and increasing the specific surface area. However, the poor conductivity impedes its direct use as SC active electrodes, limiting its practical application in energy storage and conversion. Therefore, the fundamental way to improve its electrochemical performance is to change the disadvantage of poor structural conductivity of MOFs. It is expected that this will become a research hotspot in applying pure MOF materials in energy storage in the future.

#### Mixed Metal MOFs

The poor conductivity of single metal MOFs limits the improvement of their electrochemical performance. Mixed metal MOFs can be synthesized by introducing other metal ions where their structures and properties can be regulated by adjusting the proportion of metal ions. They can offer richer redox reactions and improve the charge transfer between metal ions so that mixed metal MOFs may have more excellent capacitive properties. According to their synthesis methods and structural characteristics, mixed metal MOFs can be divided into many kinds. The following have a brief introduction to the application of bimetallic MOFs in super electrical appliances [98].

**Fig. 6:**
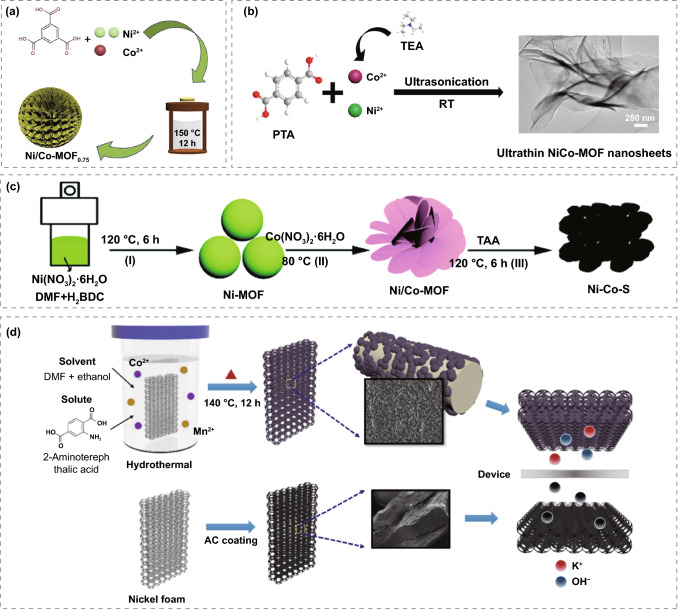
**a** Schematic diagram of the synthesis process. Reprinted with permission from Ref. [99]. Copyright 2020 Elsevier B.V. **b** Preparation technology of NiCo-MOF. Reprinted with permission from Ref. [100].Copyright 2019 American Chemical Society. **c** Schematic diagram of Ni/Co-MOF preparation process. Reprinted with permission from Ref. [102]. Copyright 2018 Royal Society of Chemistry. **d** Schematic diagram of the preparation of bimetallic Co/Mn-MOF and activated carbon. Reprinted with permission from Ref. [104]. Copyright 2019 Elsevier Ltd

The main reason for many scholars to investigate nickel–cobalt bimetallic MOFs is that cobalt-based MOFs and nickel-based MOFs as electrode for supercapacitors exhibit excellent properties. Zhang et al. [99] synthesized 2D Ni/Co-MOF nanosheets using a one-step hydrothermal method. It can be seen from XRD that the diffraction peaks of Ni/Co-MOF_0.75_ and Ni/Co-MOF_1.5_ are weaker than those of pure Ni MOF, indicating that the topology damage caused by the successful replacement of Ni^2+^ by Co^2+^ (Fig. 6a). By combining mixed metal and unique two-dimensional structure, the electrode showed excellent recycling ability of 568 C g^−1^ at 1 A g^−1^. The energy density of 42.24 Wh kg^−1^ can be achieved for supercapacitor composed of Ni/Co-MOF and reduced graphene oxide (RGO) when the power density was 800 W kg^−1^. Wang et al. [100] prepared ultrathin Ni/Co-MOF nanosheets with terephthalic acid as a ligand by simple ultrasonic treatment at room temperature and used them as electrode materials for supercapacitors (Fig. 6b). The unique nanosheet-like structure of Ni/Co-MOF offers more active sites and shortens the electron transfer paths, which makes it deliver an excellent electrochemical performance, the specific capacitance is 1202.1 F g^−1^ when the current density is 1 A g^−1^. In addition, a power density of 562.5 W kg^−1^ can be exhibited for Ni/Co-MOF//activated carbon asymmetric supercapacitors assembled in 2 M KOH electrolytes, at the energy density of 49.4 Wh kg^−1^. Hollow Spherical Ni/Co-MOF composed of nano-lamellae was synthesized by the one-step hydrothermal method with 1,2,3-propanedicarboxylic acid as organic ligand [101]. The nano-lamellae with a two-dimensional structure can offer more active sites. The hollow spheres with the three-dimensional structure are conducive to ion transfer, improving electrochemical properties. The specific capacity of 758.8 C g^−1^ can be achieved at a current density of 0.5 A g^−1^ for Ni/Co-MOF, and at 20 A g^−1^, the specific capacity is still as high as 584 C g^−1^. However, after 5000 cycles, the capacity is still 84.6% of the initial value. Chen et al. [102] reported Ni/Co-MOF nanosheets with petal-like structures synthesized by etching Ni-MOF spheres in Co (NO_3_)_2_ solution (Fig. 6c). The specific capacitance of 1220.2 F g^−1^ at 1 A g^−1^ can be achieved for Ni/Co-MOF-5, which was synthesized via modulating the proportion of Co(NO_3_)_2_ and other conditions. And the capacitance retention is 87.8% after 3000 cycles, showing good rate performance and cycle stability. Xu et al. [103] prepared Ni/Co-MOFs with different ratios by liquid-phase synthesis at room temperature. Ni/Co-MOFs with different Ni/Co molar ratios exhibit different electrochemical activities. The specific capacity of 1074.5 C g^−1^ at 1 mA cm^−2^ and 827 C g^−1^ at 20 mA cm^−2^ can be achieved for Ni_2_Co-MOF, showing remarkable charge storage capacity. In addition, the hybrid supercapacitor with Ni_2_Co-MOF as a positive electrode and activated carbon as a negative electrode provides superior energy densities of 66.1 and 41.3 Wh kg^−1^ at power densities of 800 and 8000 W kg^−1^, respectively. These results indicate the great potential of such rapidly synthesized Ni/Co-based MOF as electrode for practical supercapacitors with high-energy and high-power densities.

In addition to Ni/Co-MOFs, other bimetallic MOFs are also good choices as electrode for supercapacitors. Seo et al. [104] synthesized Co/Mn-MOF on the foamed nickel. The bimetallic MOF produced a synergistic effect mainly through organic ligands connecting two kinds of metal ions, which contributes to large specific surface area, good porosity. And direct growth of nanoparticles on the collector can reduce resistance and provide ample room for ion diffusion (Fig. 6d). When the current density is 3 mA cm^−2^, the specific capacitance of the bimetallic Co/Mn MOF electrode is 1176.59 F g^−1^ (2.76 F cm^−2^) with good cycle stability in 5000 cycles. When the specific power of the Co/Mn-MOF//AC composite supercapacitor is 2000 W kg^−1^, the specific energy is 57.2 Wh kg^−1^, and the capacity retention rate is 93.51% after 5000 cycles. Kang et al. [105] synthesized Ni/Mn-MOF by solvothermal reaction using 1,4-phthalic acid as organic ligand, nickel nitrate hexahydrate, and manganese nitrate tetrahydrate as central metal ions. The prepared Ni/Mn-MOF have a coral-like microstructure and layered pore size, with a specific surface area of 380.8 m^2^ g^−1^ and average pore size of 13.6 nm. In 6.0 M KOH electrolyte, the specific capacitance of 531.5 and 317.9 F g^−1^ for coral Ni/Mn-MOF electrode can be achieved at 0.5 and 5 A g^−1^, respectively; After 2300 cycles at 5 A g^−1^, the capacitance retention rate is 67.7%, which has high specific capacitance and good cycle stability. These results indicate that coral-like Ni/Mn-MOF is favorable for electrolyte permeation and ion migration. Kazemi et al. [106] prepared Co/Mn MOFs with different amounts of cobalt chloride (0.02, 0.04, and 0.08 g) using terephthalic acid as organic ligand and recorded as MOF-1, MOF-2, and MOF-3, respectively. The maximum specific capacitance of the MOF-2 electrode is 2.375 F cm^−2^, and the capacitance retention rate is more than 85% after 3000 times of continuous charge and discharge. The value of Ru was considerably small (almost 0.65 Ω), and *R*_ct_ was about 0.17 Ω, implying that the kinetics of electron/charge transfer at the MOF-2 electrode is significantly facilitated. Rajak et al. [107] synthesized a new nanorod-like Na_2_Co-MOF by slow diffusion technique at room temperature. The crystal structure study demonstrates that the acquired coordination network reveals as a 3D architecture when the proportion of Co(II) and Na(I) metal nodes is 1:2. Due to the incorporation of sodium ions and the synergistic effect of mixed ligands, the specific capacitance of 321.8 F g^−1^ for Na_2_Co-MOF can be achieved at 4 A g^−1^, a specific capacitance retention rate of 78.9% can be maintained at 16 A g^−1^, and 97.4% after 5000 cycles, showing excellent rate performance and cycle stability. The flower-like structures of Co/Ni-MOF and Zn/Ni-MOF were obtained by partial supersession of Ni-MOF with Co^2+^ and Zn^2+^ in Jiao's report [108]. The capacitances of 236.1 and 161.5 mAh g^−1^ can be achieved at 1 A g^−1^ for them, which are higher than those of Ni-MOF without substitution (142.31 mAh g^−1^). At a high current density of 10 A g^−1^, their capacitances are 195.6 and 90.3 mAh g^−1^, respectively, which are also higher than the 50.8 mAh g^−1^ of Ni-MOF. After 5000 cycles, the morphologies and structures of them did not show obvious changes. In contrast, the morphologies of Ni-MOF materials changed obviously, indicating that the MOF composed of two central metal ions can provide special structural stability through interaction, which is beneficial to alleviate the structural damage caused by volume expansion during the cycle.

### MOF Composite Materials as Electrode

MOF composite electrode materials are electrode materials prepared by blending MOF materials with carbon-based materials or other metals, conductive polymers, etc. Although MOFs have good electrochemical energy storage performance, the stability of structures and conductivity impede their practical application in energy storage and conversion. MOF composites not only combine the advantages of MOFs, such as structural adjustability, flexibility, high porosity, and ordered pore structure but also effectively improve the internal electron transfer rate of the composites due to the introduction of new doping components, which makes up for the shortcomings of MOFs.

#### MOFs/Carbon Composite

The carbon material is characterized by a large specific surface area, high conductivity, and good chemical stability, but it has the disadvantages of low specific capacitance and energy density. Therefore, we composite MOFs with carbon materials, improving MOFs' conductivity and dispersion. The composites of the two materials can synergize and obtain better capacitive properties than single materials. These carbon materials have unique hollow structures and nano-sized mesh structures that can be intertwined and have excellent properties such as good conductivity, high contactable surface area, and good chemical and thermal stability. However, CNTs cannot meet the actual needs because of their low specific surface area, small specific capacitance, serious self-discharge, and easy agglomeration. Combining it with MOFs can effectively improve these problems. Carbon nanofibers have large specific surface areas and great conductivity, while graphene also has excellent electrochemical properties with a theoretical capacitance of 550 F g^−1^ making it an ideal electrode material.

**Fig. 7:**
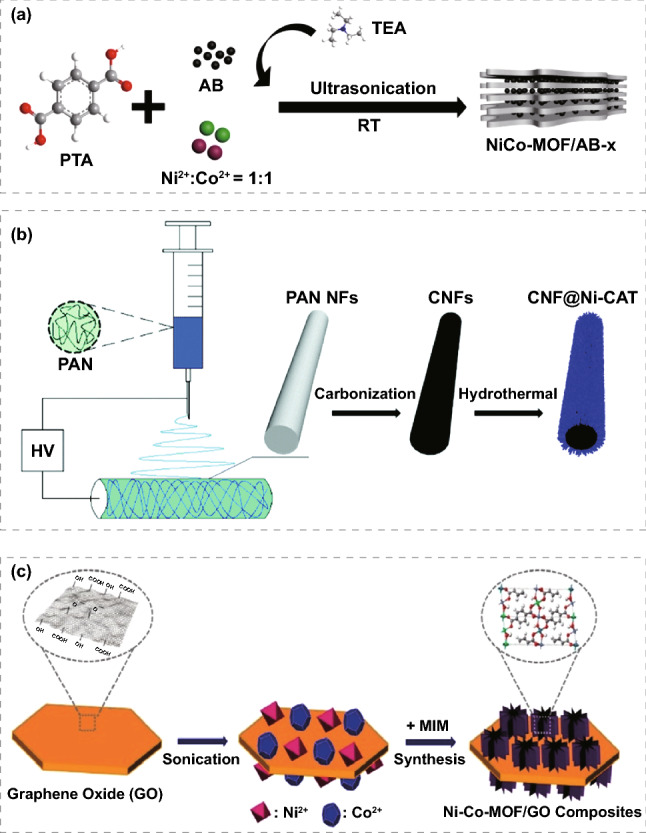
**a** Schematic diagram of the synthesis of NiCo-MOF/AB composites. Reprinted with permission from Ref. [112]. Copyright 2019 Elsevier B.V. **b** Synthesis diagram of CNF@Ni-CAT. Reprinted with permission from Ref. [113]. Copyright 2019 Royal Society of Chemistry. **c** Preparation process diagram of Ni-Co-MOF/graphene oxide composites. Reprinted with permission from Ref. [114]. Copyright 2019 Elsevier Ltd

Wen et al. [109] synthesized the Ni-MOF@CNT by directly growing Ni-MOF on the surface of CNTs. In the Ni-MOF/CNTs composites, CNTs were uniformly wrapped by Ni-MOF sheets. The specific capacitance of 1765 F g^−1^ for Ni-MOF/CNTs can be achieved at 0.5 A g^−1^, which is 1.6 times of 1080 F g^−1^ for pure Ni-MOF. In addition, after 5000 charge and discharge tests, Ni-MOF@CNT still retains 95% of the capacitance. This predominance can be attributed to the synergistic effect of Ni-MOF and CNTs. Ni MOF has a porous structure, which can promote the entry of electrolytes, while CNT can effectively improve the conductivity of the whole composite capacitor. Zhang et al. [110] first mixed CNTs with Mn(C_2_H_4_O_2_)_2_(H_2_O)_4_ in solution, then added the ligand (NH_4_)_2_C_8_H_4_O_4_ and stirred it to synthesize CNTs@Mn-MOF and then investigated their electrochemical behaviors as supercapacitor cathodes. CNTs provide strong mechanical support and conductive network for the MOF composite, leading to the intrinsic improvement of conductivity and the inherent increase in specific capacitance. At the same time, after 3000 times of repeated charge and discharge tests, the capacity retention of the CNTs@Mn-MOF electrode is 88%. This research lays a foundation for developing Manganese-based electrode materials and provides a useful method to improve the capacitance performance of MOFs electrodes. Srimuk et al. [111] composited graphene oxide (rGO) with Cu-MOF (HKUST-1) to improve the conductivity of MOF. rGO/HKST-1 inherits the high specific surface area and high porosity characteristics of Cu-MOF, the BET specific surface area of which is 1241 m^2^ g^−1^, and average pore size is 8.2 nm. It can provide a suitable pore size for the absorption and release of electrolytes, leading to an excellent supercapacitor performance. The specific capacitance of 385 F g^−1^ can be achieved for it at 1 A g^−1^, which of Cu-MOF without composite rGO is only 0.5 F g^−1^. The NiCo-MOF/acetylene black composite was prepared by ultrasonic method [112]. Acetylene black was evenly distributed among NiCo-MOF nanosheets, which hindered their aggregation. This unique structure can offer a higher specific surface area for redox reaction, shorten the distance of electron transfer and ion diffusion, and significantly improve the capacity of NiCo-MOF compared with pure NiCo-MOF. The NiCo-MOF/acetylene black composite exhibits a high specific capacitance of 916.1 F g^−1^, remarkable rate performance, and cycle stability, which is 85.25% of the initial capacitance after 5000 cycles (Fig. 7a). Zhao et al. [113] combined electrospinning technology with the hydrothermal method to synthesize a core–shell composite material using carbon nanofibers and two-dimensional conductive MOFs. The Ni-CAT nanorods uniform grown on the surface of CNFs by electrospinning not only solves the agglomeration problem of Ni-CAT nanorods but also greatly increases the contact area between the composite and electrolyte. In addition, the overall one-dimensional properties of CNFs can effectively accelerate the electron transfer. As electrode materials for supercapacitors, CNF@Ni-CAT delivers a high specific capacitance of 502.95 F g^−1^ at 0.5 A g^−1^. Due to its excellent synergistic effect, its cycle stability after 5000 cycles is improved by 73%. When the power density is 297.12 W kg^−1^, the energy density of 18.67 Wh kg^−1^ for the supercapacitor composed of CNF@Ni-CAT and AC is achieved. After 5000 cycles, the specific capacitance of 106.19% to the original specific capacitance can be maintained (Fig. 7b). Jihwa et al. [114] prepared mixed MOFs with a constant proportion of Ni^2+^ instead of Co^2+^. The conductivity of MOFs can be improved without calcination due to the addition of nickel with good conductivity. By adding graphene oxide to Ni/Co-MOF, the charge capacitance of Ni/Co-MOF@GO composite was further improved. By controlling the amount of graphene oxide in Ni/Co-MOF, Ni/Co-MOF@GO_2_ (the amount of graphene oxide is 0.2 g) exhibits the highest energy storage performance (Fig. 7c). When the current density is 1 A g^−1^, the maximum specific capacitance is 447.2 F g^−1^ and the life stability of Ni/Co-MOF@GO_2_ is 99.6% after 300 cycles. The graphene oxide nickel–cobalt metal–organic framework complex with a 3D chestnut shape delivers high capacitance, stable charge/discharge capacity, good conductivity, and long cycle life, which is expected to pave the way for the development of supercapacitor materials with good electrochemical performance. Chen et al. [115] directly grown porous Ni/Co-MOF with honeycomb structure on carbon cloth by hydrothermal method (Ni/Co-MOF@CC). The honeycomb porous structure promotes the interaction between the active material and electrolyte, shortens the electron transfer path, and improves the active sites of electrochemical reactions. The nanosheets do not need any adhesive to be directly deposited on the framework of carbon cloth, so the Ni/Co-MOF@CC has a high specific surface area, an average pore size of 3.05 nm, and excellent conductivity. The electrochemical performance of porous Ni/Co-MOF@CC as supercapacitor electrode in 2 M KOH electrolyte was evaluated. The maximum specific capacity of 1180.5 mC cm^−2^ can be achieved at 3 mA cm^−2^ for Ni/Co-MOF@CC electrode, 624.1 mC cm^−2^ at 60 mA cm^−2^ and 97.6% at 5000 cycles. The results show that the M-MOF@CC electrode has great potential for applying the MOF family in the area of electrochemical energy storage. Liu et al. [116] prepared a supercapacitor with synergistic interaction between growth-oriented iron-based MOF and GA composites. MIL-88-Fe was grown in situ on the (002) lattice surface of graphene by one-step solvothermal method. A strong P-π interaction can be produced between the graphene sheets and MOFs because of the long period hexagonal structure and electrophilicity. The existence of MOF can influence the electric double-layer characteristics of composites by utilizing the rich space of GA which results in the advantages of large capacitance, fast charging and discharging rate and good cycling stability. Specific capacitance up to 353 F g^−1^ with a scan rate of 20 A g^−1^. Fischer et al. [117] reported covalent assembly of GA with amine functionalized MOF (UIO-66-NH2). It shows the characteristic of graded porosity. The micropores were derived from octahedral UIO-66-NH2 nanocrystals, the mesopore is derived from the distance between GA-GA layers, which is formed by covalent bonding between GA and MOF spacer via amide bridge. In addition, it is expected that their covalent assembly will confer new properties, including graded pores for fast ion transport, accessible interaction sites, and conductive networks for interconnection, all of which benefit the application of supercapacitors. Electrochemical tests show that hybrid GA@UiO-66-NH2 is an effective charge storage material with a capacitance up to 651 F g^−1^, significantly higher than conventional graphene-based materials.

#### MOFs/Conductive Polymer Composite

Conductive polymers, including polypyrrole (PPY), polyaniline (PANI), polythiophene (PTH), and their derivatives, have aroused a great deal of research interest because of their good conductivity and electrical activity. Polyaniline is a common π conjugate conductive polymer material, which is often used as MOF composites because of its simple synthesis method, good electronic conductivity, and appropriate chemical stability.

**Fig. 8:**
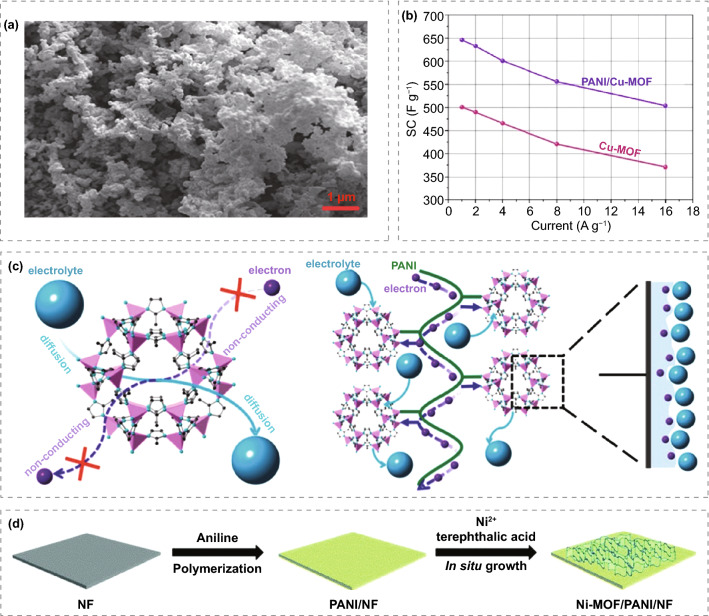
**a** SEM of PANI/Cu-MOF nanocomposite. **b** Specific capacitance of Cu-MOF and PANI/Cu-MOF. Reprinted with permission from Ref. [118]. Copyright 2019 Springer Science Business Media. **c** Schematic diagram of electron conduction in MOF and MOF interweaves. Reprinted with permission from Ref. [119]. Copyright 2015 American Chemical Society. **d** Schematic diagram of Ni-MOF/PANI/NF synthesis. Reprinted with permission from Ref. [121]. Copyright 2019 Royal Society of Chemistry

PANI/Cu-MOF was prepared by chemical polymerization of aniline and synthesis of Cu-MOFs at room temperature (Fig. 8a) [118], which has a hemispherical structure similar to polyaniline. The composite exhibits pseudocapacitance behavior in 6 M KOH electrolyte, and the specific capacitance (734 F g^−1^) is higher than that of pure Cu-MOF (558 F g^−1^) (Fig. 8b). It is believed that polyaniline increases the active sites in the redox process by providing pathways for electron transfer and ion channels, thus improving the specific capacitance of PANI/Cu-MOF. The capacitance of Cu-MOF and PANI/Cu-MOF composites remained about 93.6% and 98% after 4000 cycles. Therefore, the preparation of Cu-MOF composites with polyaniline as the conductive polymer is an effective method to improve the cycle life and specific capacitance of Cu-MOF. Wang et al. [119] interweaved MOFs with polyaniline (PANI) chains, which cannot only effectively increase the conductivity but also promote the faraday process of the interface. Specifically, Cobalt-based MOF crystal (ZIF-67) was synthesized on carbon cloth (CC). Then polyaniline was electrodeposited to obtain a flexible conductive porous electrode (PANI-ZIF-67-CC) without changing the structure of MOF. Electrochemical studies showed that a specific capacitance of 2146 mF cm^−2^ for PANI-ZIF-67-CC can be achieved at 10 mV s^−1^. This is because polyaniline chains not only cover the surface of MOF but also connect the isolated MOF crystals and become the bridge between MOF particles. PANI long chains and MOF are cross-linked, which can transfer the external electrons to the internal MOF surface and effectively improve the material's conductivity (Fig. 8c). Rajkumar et al. [120] prepared Co-MOF/PANI composites by in situ oxidative chemical polymerization using cobalt nitrate, 1,3,5-phenyltricarboxylic acid, and aniline as raw materials. The Co-MOF/PANI composite shows a closely connected network structure, which helps to stabilize the structure of PANI and prevent the structure collapse of the composite. This unique structure can also provide a fast ion transport path for the electrode electrolyte interface, resulting in better supercapacitor performance. It is found that the capacitance of Co-MOF/PANI composite is enhanced when the scanning rate is 5 mV s^−1^. The specific capacitance of 504 F g^−1^ for it can be achieved at 1 A g^−1^. After 5000 cycles at 2 A g^−1^ current density, the specific capacitance is still 90% of the initial capacitance. The results show that Co-MOF/PANI composite can be used as potential electrode material for energy storage devices. Cheng et al. [121] has primarily reported that ultra-thin Ni-MOF nanosheets arrays, which can be used as a self-supporting binder free electrode for supercapacitors, are directly grown on polyaniline modified nickel foams (Fig. 8d). Polyaniline improves the conductivity of Ni-MOF nanosheets and accelerates the formation of Ni-MOF nanosheets array, ensuring excellent mechanical adhesion. The higher area capacitance of 3626.4 mF cm^−2^ for Ni-MOF/PANI/NF can be achieved at 2 mA cm^−2^ and better rate capacitance of 71.3% can be maintained at 50 mA cm^−2^. In addition, the energy density of 45.6 Wh kg^−1^ for the asymmetric supercapacitor with Ni-MOF/PANI/NF and AC can be achieved, and the capacitance retention rate of 81.6% can be exhibited after 10,000 cycles. Ni-MOF sheets with different ratios of polypyrrole (0.05, 0.1, 0.15, 0.2, or 0.3 g) were synthesized via a simple wet chemical method by Wang et al. [122]. Scanning electron microscopy showed that the surface of Ni-MOF sheet was covered with PPY, and the overall morphology of Ni-MOF did not change significantly, proving that the introduction of PPY did not break the structure of Ni-MOF. The ppy-MOF composite was used as electrode material of supercapacitor. The electrochemical results show that the capacitance of 715.6 F g^−1^ can be achieved at 0.3 A g^−1^ for ppy-MOF_0.2_ and 449.5 F g^−1^ at 8 A g^−1^. Which prove that ppy-MOF_0.2_ composite has good rate performance. In addition, an asymmetric supercapacitor, which can deliver a high energy density of 40.1 Wh kg^−1^ at a power density of 1500.6 W kg^−1^, and capacitance of 398.2 F g^−1^ at 0.2 A g^−1^, was assembled with ppy-MOF and activated carbon as positive and negative electrodes.

#### MOFs/Metals

**Fig. 9:**
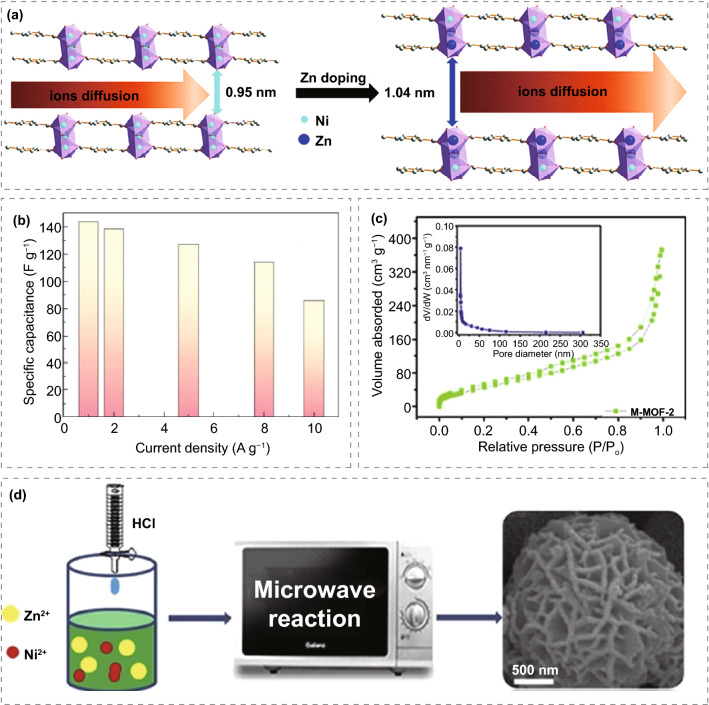
**a** Possible structural changes of Ni-MOF before and after Zn doping. Reprinted with permission from Ref. [123]. Copyright 2014 Royal Society of Chemistry. **b** Specific capacitance at different current densities. Reprinted with permission from Ref. [124]. Copyright 2020 Elsevier Ltd. **c** BET of Ni-MOF. **d** A Zn-doped Ni-MOF with honeycomb layered spherical structure by the microwave-assisted synthesis method. Reprinted with permission from Ref. [126]. Copyright 2020 Wiley–VCH GmbH

Different from conductive polymers, metal doping can effectively improve the structural stability of MOFs and improve the electrochemical properties. Yang et al. [123] synthesized a zinc-doped layered Ni-MOF and then as electrode for supercapacitors. The layered structure can supply enough room for electrolyte diffusion during circulation, and Zn^2+^ doping can prevent crystal collapse (Fig. 9a). Moreover, the Ni-MOF doped with Zn^2+^ shows a lower charge transfer resistance, which indicates that it has a faster electron transfer rate. It is believed that the powder microsphere structure can provide open pores for electrolyte diffusion, and the porous spherical structure can prominently buffer the volume change caused by the circulation. For these reasons, the supercapacitor exhibits the characteristics of large specific capacitance, high magnification, and good cycle stability. At 10 A g^−1^, the capacitance of 854 F g^−1^ can be achieved. At the same time, the retention rate remained above 91% even after 3000 cycles. Xiong et al. [124] has designed a new strategy in which NiO is used as a new self-sacrificing template and precursor to growing on foamed nickel (NF) (Fig. 9b). After adding an organic ligand (H_3_BTC), it is gradually transformed into a Ni-MOF array. Finally, the porous cylindrical cage-like structure NiO@Ni-MOF/NF is successfully prepared. The process is simple and easy to operate without further template removal. This unique structure can enhance the conductivity of the electrode and make it have a good orientation, which can promote the transfer of electrons and ions resulting an excellent electrochemical performance for NiO@Ni-MOF/NF. The specific capacity of NiO@Ni-MOF/NF-12 is 1853 C cm^−2^ (1 mA cm^−2^), which is better than NiO/NF (242 C cm^−2^), and the impedance is relatively low. The hybrid supercapacitor (HSC) uses carbon nanotube (CNT) as the negative electrode and NiO@Ni-MOF/NF-12 as the positive electrode. The specific capacitance of 144 F g^−1^ for HSC can be achieved at 1 A g^−1^. After 3000 cycles, the capacitance of 94% can be maintained. A novel Zn doped Ni-MOF with honeycomb layered spherical structure was synthesized by Chen et al. [125] using HCl as a modulator for the first time (Fig. 9c, d). The optimal Zn doped Ni-MOF electrode material was obtained by adjusting the amount of Zn ion doping. The electrochemical performance of 237.4 mAh g^−1^ for M-MOF-2 can be achieved at 1 A g^−1^, and the specific capacity retention rate of 88% can be maintained after 4000 cycles. Its excellent electrochemical performance is attributed to (1) its porous honeycomb structure promoting electrolytes' diffusion. (2) The introduction of larger Zn increases the interlayer distance of MOF, provides enough space for the diffusion of electrolyte ions, and can also be used as a support column to prevent the crystal collapse during the cycle. However, too much or too little zinc ion doping will lead to the aggregation or formation of small particles, which impedes the transfer of charges from active electrode to the electrolyte and the rapid diffusion of electrolyte ions. (3) Acidic synthesis environment is conducive to the formation of microspheres and improving crystallinity and electrochemical performance. This rapid microwave-assisted synthesis method has a good application prospect in preparing battery supercapacitor electrode materials. From Zheng's report [126], thin Ni-MOF nanosheets doped with Mn were designed on foamed nickel as high capacitance and stable supercapacitor positive electrode (Mn_0.1_-Ni-MOF/NF). In the 6.0 M KOH solution, the specific capacity of 1178 C g^−1^ is achieved for Mn_0.1_-Ni-MOF/NF at 2 mA cm^−2^, which is larger than all MOF-based materials. More importantly, it has good cycle stability and maintains 80.6% capacity after 5000 cycles. The energy density of 39.6 Wh kg^−1^ can be achieved for the device with Mn_0.1_-Ni-MOF/NF and activated carbon, when the power density is 143.8 W kg^−1^, and the capacitance retention is 83.6% after 5000 cycles. This is because Manganese has a variety of valence states, such as Mn^2+^, Mn^3+^, and Mn^4+^. This redox characteristic is conducive to the overall charge storage capacity in the charge/discharge process under alkaline conditions. The poor conductivity of Ni(OH)_2_ and NiOOH results in the difficulty of charge transport in the materials. Mn doping can effectively adjust the electronic density of states of Ni, which greatly promotes the charge transfer compared with the undoped structure.

### MOFs-Derived Materials as Electrodes

In recent years, MOFs as precursors or templates, through simple inert gas pyrolysis, air annealing, microwave irradiation or solution dissolution, synthesis of porous carbon [127], metal sulfide [128–131], and oxide as electrode materials for supercapacitors have become one of the research hotspots. The derivatives prepared mainly in this way can largely maintain the characteristic of the precursor MOFs, including high specific surface area, large pore volume, and even special skeleton structure. These advantages make MOF derivatives attract much attention in the field of SCs.

#### MOFs-Derived Carbon Materials

**Fig. 10:**
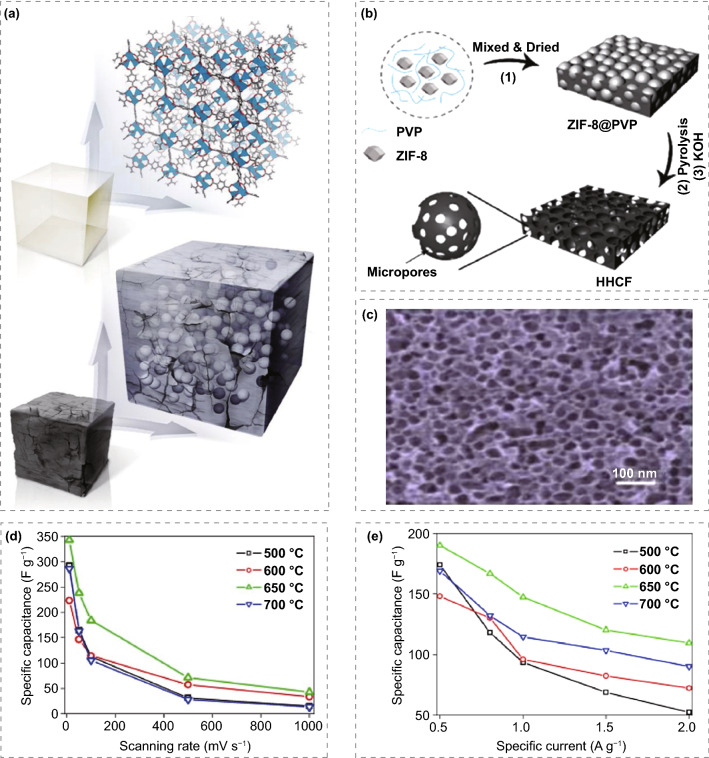
**a** Schematic synthesis diagram of IRMOF-1 (top) and carbon-coated ZnO QDs without agglomeration produced by IRMOF-1 after controlled pyrolysis (bottom). Reprinted with permission from Ref. [134]. Copyright 2013 American Chemical Society. **b** HHCF synthesis process diagram. **c** SEM images of HHCF. Reprinted with permission from Ref. [136]. Copyright 2019 Elsevier B.V. **d** Specific capacitance with respect to scan rate. **e** Specific capacitance as a function of current density. Reprinted with permission from Ref. [138]. Copyright 2018 Elsevier Ltd

Carbon materials are widely employed in supercapacitors, especially electric double-layer capacitors, due to their large surface area and good stability [132]. But its specific capacitance is small, so it is difficult to improve the power density and energy density. Preparing microporous/mesoporous carbon materials with unique structures, using MOFs as templates, has attracted extensive attention. The prepared porous carbon materials maintain the high specific surface area and uniform pore size distribution of the precursor MOFs to a great extent, which is favorable for the effective contact between carbon materials and electrolytes and the improvement of their electrochemical performance. Xu et al. [133] reported for the first time in 2008 that carbon materials from MOF precursors with a large comparative area and excellent electrochemical properties which introduced furfuryl alcohol as carbon source to improve the porosity. The temperature-programmed method was used for the polymerization process and subsequent carbonization process of FA. When it is carbonized in Ar at 1000 °C, the organic components are cracked through the carbonization process, and Zn is taken away by argon due to gasification (the boiling point of Zn is 907 °C), leaving a carbon skeleton with high porosity. The BET specific surface area of the obtained nanoporous carbon (NPC) is 2872 m^2^ g^−1^, and the pore size is from micropore to macropore. The capacitance of 258 F g^−1^ can be achieved at 250 mA g^−1^. The structure change process of porous carbon prepared by carbonizing Zn-MOFs at different temperatures was studied [134]. The results show that MOFs can still maintain their skeleton structure when the temperature is lower than 550 °C. With the further increase in temperature, MOFs structure gradually decomposes into C and ZnO, and ZnO is reduced to Zn by the carbon generated in the system. When the temperature is increased to 908 °C, Zn is lost at the high boiling point. Therefore, high purity carbon can be synthesized effectively at higher temperatures (Fig. 10a). Yan et al. [135] directly prepared porous carbon at 800 °C using three porous materials, HKUST-1, MOF-5 and Al-MOF, as templates and carbon sources. The BET results show that the specific surface areas of the three MOFs derived from porous carbons are 50, 420, and 1103 m^2^ g^−1^, respectively. The carbon obtained from HKUST-1 exhibits the lowest specific surface area and poor electrochemical stability. MOF-5 and Al-MOF-based carbon show good performance for electrodes. The results show that the porous carbon prepared by Al-MOF exhibits the best electrochemical performance, which is 232.8 F g^−1^ at 0.1 A g^−1^. The main reason might be that this kind of porous carbon inherits the large specific surface area and special pore structure of MOFs, which is conducive to charge transfer and ion transfer. Deng et al. [136] reported the hierarchical porous honeycomb carbon skeleton (HHCF) preparation by pyrolysis of metal–organic skeleton complex and activation. HHCF has a high surface area and many micropores and mesopores introduced in the activation process to provide more adsorption sites for electrolyte ions, promote the rapid transport of ions, and improve the electrolyte permeability (Fig. 10b, c). In water-electrolyte, the specific capacitance of 361 F g^−1^ can be achieved at 1 A g^−1^ for the HHCF electrode and 182 F g^−1^ at 100 A g^−1^. In addition, the symmetrical super-capacitor exhibits a specific capacitance of 174 F g^−1^ at 1 A g^−1^ and an excellent energy density of 74 Wh kg^−1^ at 872 W kg^−1^. The above studies show that MOFs are perfect templates for preparing porous carbon materials. For carbon supercapacitors, the microporous structure can provide high capacitance, while mesoporous and macroporous are conducive to the magnification performance of capacitors. In addition, the specific surface area and carbonization temperature of electrode materials will also affect the capacitance. Li et al. [137] successfully synthesized ordered macro-microporous single-crystalline MOF with a spatially constrained growth model. The carbon material derived from it inherits the ordered interconnecting macroporous structure. When used as electrode for supercapacitors, the improved diffusivity and low resistance as well as structural robustness give the derived carbon materials excellent rate performance and excellent cycle stability. It is expected that this method will provide a new way to synthesize such macroporous and microporous materials for energy related fields and other fields.

The carbon polyhedron and carbon nanotube hybrid (HCN) were synthesized using ZIF-67 as precursor [138]. Firstly, uniform ZIF-67 polyhedral nanocrystals were prepared, and then carbon nanotubes were grown by chemical vapor deposition. HCN shows a typical network structure in which ultra-long carbon nanotubes tightly connect polyhedral porous carbon. By investigating the effect of growth temperature on HCN, it was found that the HCN (HCN-650) electrode synthesized at 650 °C exhibited remarkable electrochemical performance; and that the highest capacitance was 343 F g^−1^ at the scanning rate of 10 mV s^−1^, and had excellent rate performance (Fig. 10d, e). Xu Qiang's team [139] mixed the MOF material and sodium hypophosphite and sealed it into a tubular furnace. Then it was heated to about 300 °C and kept at this temperature for two hours before further heating to high temperature to complete carbonization. At 300 °C, the sodium hypophosphate would decompose gradually to produce phosphine gas. Under such atmospheric conditions, phosphine gas would selectively corrode different crystal surfaces of MOF. Interestingly, the method "fumigates" the MOF directly under solid state conditions without using any organic solvents, resulting in the hetero-atom doped porous carbon cage material with openings in the wall, displaying high efficiency based on zinc ion energy storage capacity and ultra-high chemical stability. In this study, the structure, morphology and properties of MOF derivatives were precisely controlled, which greatly expanded the application of MOF-based materials in the field of electric energy storage.

#### MOFs-Derived Metal Oxides

Supercapacitors based on transition metal oxides store energy mainly through Faraday charge transfer generated by redox reaction during charging and discharging [140, 141]. The Faraday pseudocapacitance of metal oxide can occur not only at the interface between the electrode and the solution but also in the inner part of the electrode itself, which greatly improves the utilization rate of the material, so the metal oxide material has a larger specific capacitance. In order to prepare transition metal oxide electrode materials with high specific surface area, high electrolyte permeability, and rich active sites, people try to synthesize porous or hollow metal oxides with MOFs as precursors. There are usually two methods to prepare metal oxides with MOF as the precursors. The first is to calcine MOFs in one step in the air to prepare corresponding metal oxides. The second is that MOFs are pyrolyzed in an inert atmosphere to obtain metal or metal oxide wrapped in carbon material and then calcined in air to obtain the corresponding oxide. Maiti et al. [142] prepared CeO_2_ nanorods based on [Ce(BTC)(H_2_O)]_*n*_ precursor (Ce-BTC). The synthesized Ce-BTC and CeO_2_ have mesoporous properties. The BET surface areas were 27.3 and 77 m^2^ g^−1^, which may be due to the unique brick to brick morphology containing a large number of voids. The faraday specific capacitance of 1204 F g^−1^ can be achieved at 0.2 A g^−1^ for it, and at a higher current density of 1.5 A g^−1^, the excellent stability of CeO_2_ is close to 100% after 5000 charge/discharge cycles.

**Fig. 11:**
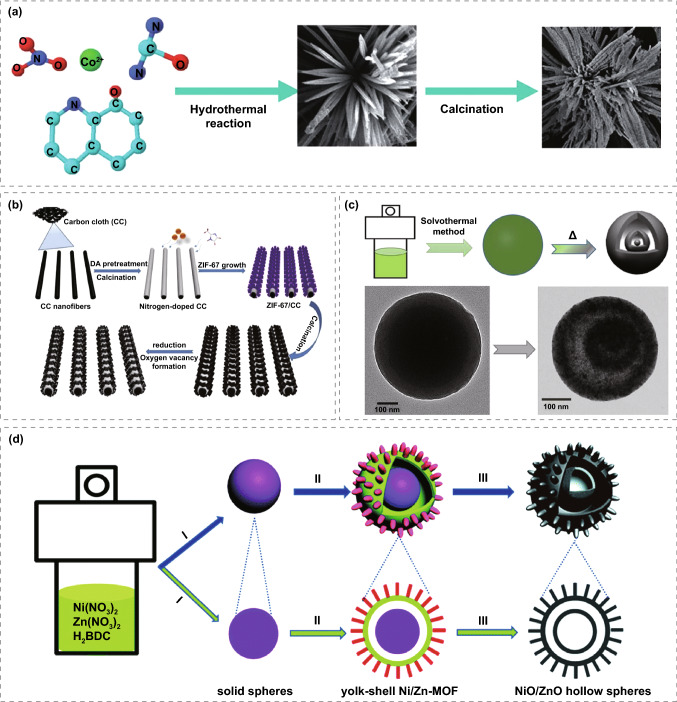
**a** Synthesis scheme of Co_3_O_4_ nanostructure. Reprinted with permission from Ref. [143]. Copyright 2012 Royal Society of Chemistry. **b** Nitrogen-doped Co_3_O_4_ nanosheets contain oxygen vacancy prepared on CC substrate. Reprinted with permission from Ref. [145]. Copyright 2019 Elsevier Ltd. **c** Diagram of synthesis of NiO nanospheres. Reprinted with permission from Ref. [147]. Copyright 2017 Elsevier B.V. **d** Schematic diagram of synthesis for NiO/ZnO hollow spheres with double shells. Reprinted with permission from Ref. [148]. Copyright 2016 Royal Society of Chemistry

Co_3_O_4_ is widely used as an electrode material due to the high theoretical capacitance of 3600 F g^−1^ and good electrochemical stability. Pang group [143] successfully synthesized Co_3_O_4_ dendrimers by calcining Co-8-hydroxyquinoline coordination precursors in air, composed of many nanorods with diameters of 15–20 nm and lengths of 2–3 μm. As the electrode material of SC, the specific capacitance of the Co_3_O_4_ nanostructured electrode at 0.5 A g^−1^ current density is 207.8 F g^−1^, which is much higher than the 77.0 F g^−1^ of commercial Co_3_O_4_ electrode (Fig. 11a). Moreover, the specific capacitance of 75.0 F g^−1^ for the Co_3_O_4_ nanostructured electrode can be achieved at 6.0 A g^−1^, which means that the material can provide high energy at high power. Zhang et al. [144] prepared porous Co_3_O_4_ by a two-step method. Firstly, they prepared ZIF-67 oblique dodecahedral microcrystals and then prepared porous Co_3_O_4_ with rhombic dodecahedral structure by calcination. The framework provides a good charge transfer platform for electrochemical reactions. The specific capacitance of 1100 F g^−1^ can be achieved at 1.25 A g^−1^ for porous Co_3_O_4_, which outclass the traditional Co_3_O_4_. Even at the current density of 6.25 A g^−1^ for more than 6,000 charge/discharge cycles, the obtained electrode exhibits a very small capacitance attenuation ratio, and the capacitance still retains 95.1%. Dai et al. [145] used Co-MOF as a self-sacrificial template to obtain ordered corrugated Co_3_O_4_ nanoarrays; first, coated the carbon cloth with a hydrophilic thin carbon layer (polydopamine derivative) to stabilize the interfacial coordination between Co_3_O_4_ and carbon fiber. Secondly, by reducing Co_3_O_4_ and introducing oxygen vacancies, the electron and ion transfer at the interface between Co_3_O_4_ and electrolyte is improved (Fig. 11b). The specific capacity of 414 C g^−1^ can be achieved at 1 A g^−1^ and the stability (0.00174% loss per cycle in 15,000 cycles) of the electrode was significantly improved. The asymmetric supercapacitors assembled with v-Co_3_O_4_/CC composite as the positive electrode and the same MOF-derived carbon nanosheets as negative electrode showed high volume and weight energy densities of 14.7 mW cm^−3^ and 45.3915 W kg^−1^, respectively.

In addition, NiO has high theoretical capacitance (more than 2500 F g^−1^) and chemical stability. Han et al. [146] successfully prepared a porous nickel oxide structure by calcining a new type of Ni-MOF in air. The obtained three-dimensional porous nickel oxide structure is assembled by two-dimensional nanosheets, which shows relatively good electrochemical performance due to its porous structure and fine nanoparticle block. The prepared NiO exhibits high specific capacitance, good rate performance, and excellent cycle stability. After 1000 cycles at 1 A g^−1^, the nickel oxide structure delivers a reversible specific capacitance of 324 F g^−1^. When the current density increases from 1 to 40 A g^−1^, its capacity retention rate is close to 46%, indicating that it has a broad application prospect in supercapacitors. By controlling the calcination temperature (400, 500, and 600 °C), Wu et al. [147] obtained highly uniform NiO hollow nanospheres with core–shell structures using Ni-MOF as precursors. NiO nanospheres calcined at different temperatures have various surface areas and conductivity, which play a significant role in the redox reaction on the surface of active materials (Fig. 11c). The results show that the hollow double-shell NiO nanospheres calcined at 400 °C exhibit the best charge storage performance. The specific capacitance of 473 F g^−1^ for it can be achieved at 0.5 A g^−1^, and the capacitance retention of 94% can be maintained even after 3000 cycles. Without adding any templates and surfactants, Ni/Zn-MOF nanorod microspheres were synthesized by solvothermal method [148]. Hierarchical double-shell NiO/ZnO hollow spheres were synthesized by calcining bimetallic organic frameworks in air. As supercapacitor electrodes, a high capacitance of 497 F g^−1^ at 1.3 A g^−1^ can be achieved for NiO/ZnO hollow spheres, representing excellent cycle stability, increasing by 17.1% after 2000 cycles (Fig. 11d). The excellent electrochemical data is believed owe to the unique double-layer NiO/ZnO hollow structure, which provides free space to adapt to the volume change during ion insertion and separation. It provides abundant electroactive sites for electrochemical reaction. The above studies show that metal oxides prepared with MOFs as templates have good electrochemical properties when used as supercapacitor electrodes. This is because the metal oxides derived from MOFs have suitable ion transport channels and large contact area, which improves the electrochemical performance. Saleki et al. [149] developed a MOFs assisted self-templating method by first synthesizing cobalt-copper-glycerol (CC-gly) spherical precursors and selecting them as metal ion sources. Subsequently, a uniform bimetallic copper-cobalt zeolite imidazolate skeleton was formed around the CC-gly spherical nucleus (ZCC-gly). Finally, the ZCC-gly yolk shell spheres were transformed into CuCo_2_O_4_ (ZCCO) double shell hollow spheres with 93 m^2^ g^−1^ specific surface area by annealing in air atmosphere. The electrode material has excellent electrochemical properties and ultra-high specific capacity of 701 C g^−1^ at 2 A g^−1^.

#### MOFs-Derived Metal Sulfides

**Fig. 12:**
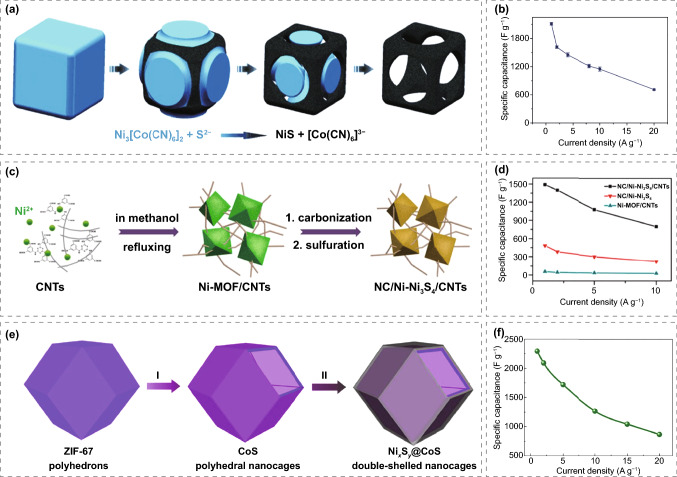
**a** Diagram of NiS nanoframe formation process. **b** The specific capacitance was calculated from the discharge curve. Reprinted with permission from Ref. [150]. Copyright 2015 WILEY–VCH Verlag GmbH & Co. KGaA **c** Scheme of the synthesis of NC/Ni-Ni_3_S_4_/CNTs composite. **d** The specific capacitances at different current densities. Reprinted with permission from Ref. [151]. Copyright 2020 American Chemical Society. **e** Schematic diagram of the formation of amorphous Ni*x*S*y*@CoS double-shelled nanocages. **f** Corresponding specific capacitance calculated from the discharge curves. Reprinted with permission from Ref. [152]. Copyright 2017 Elsevier Ltd

As one of the electrode materials with high-performance pseudocapacitance, transition metal sulfide (TMS) has attracted much attention due to its better conductivity and electrochemical activity than traditional electrode materials. The transition metal sulfides prepared with MOFs as precursors can form a variety of adjustable morphology, such as nanoparticles, nanowires, nanotubes, and nanosheets, improving the conductivity and shortening the diffusion of electrons ions paths, thus enhancing the performance of supercapacitors. Yu et al. [150] reported the preparation of shape adjustable NiS hollow cages from cubic Ni-Co PBA (Prussian blue analogue) synthesized by MOFs. S_2_ continuously etched the edge of the Ni-Co PBA cube. When the reaction time reached 6 h, the NiS with a hollow cage structure could be synthesized. Its open frameworks, high specific surface area, and good structural stability are very conducive to the electrochemical energy storage of NiS as electrodes. The results from electrochemical charge/discharge tests show that NiS has a high specific capacitance. The specific capacitance of 2212 F g^−1^ for it can be achieved at 1 A g^−1^, and the specific capacitance can be retained by 91.8% after 4000 cycles of charge/discharge. This may be because that there are many defects and active sites on the edge of the special framework of the NiS hollow cage, which is very conducive to the redox reaction, thus making it have a strong capacitance output capacity (Fig. 12a, b).

Yang et al. [151] used nitrogen-rich functional group ligands as raw materials to construct Ni-MOF and then compounded them with carbon nanotubes to prepare NC/Ni-Ni_3_S_4_@CNTs composites. Due to the construction of a three-dimensional conductive network and the introduction of nitrogen doping, the conductivity is improved, the rapid entry of electrolyte is promoted, and the reaction kinetics of NC/Ni-Ni_3_S_4_@CNTs is enhanced, and the excellent specific capacitance, coulomb efficiency, and cycle stability are obtained. The specific capacitance of 1489.9 F g^−1^ can be achieved at 1 A g^−1^ for NC/Ni-Ni_3_S_4_@CNTs (Fig. 12c, d). Gao et al. [152] chemically transform ZIF-67 polyhedron into hollow cos nanocage, and then amorphous Ni_*x*_S_*y*_ nanoparticles grow on the 3D framework of CoS to form the Ni_*x*_S_*y*_ double shell. Polyhedron nanocage shells can be easily adjusted to adapt to various combinations. High specific capacitance (2091 F g^−1^ at 2 A g^−1^) and excellent cycle stability (85.2%, cycle 2000, 5 A g^−1^) were obtained (Fig. 12e, f). Hou et al. [153] first prepared the ZIF-67 template from Co(NO_3_)_2_·6H_2_O. Later, ZIF-67 was transformed into Co_3_S_4_ by a simple hydrothermal method using thioacetamide (TAA) as a sulfur source, and NiO nanosheets were grown on the ZIF-67 surface to prepare hollow dodecahedral Co_3_S_4_@NiO. The hollow structure of Co_3_S_4_@NiO exposes more active sites, promotes the free diffusion of electrolyte, shortens the path of electron transfer in the electrochemical reaction process, and the transition metal oxide NiO grows on Co_3_S_4_ forms a shell. Both participate in energy storage, which is conducive to improving the storage capacity. NiO nanosheets on the surface of Co_3_S_4_ can also buffer the volume effect during the charge/discharge process and improve the stability of the material. Therefore, Co_3_S_4_@NiO exhibits high specific capacitance (1877.93 F g^−1^ at 1 A g^−1^) and excellent cycle stability (92.6% capacitance retention after 10,000 cycles). Asymmetric supercapacitor (Co_3_S_4_@NiO//AC) is assembled with Co_3_S_4_@NiO as positive electrode and activated carbon as negative electrode, where the power density is 0.78 kW kg^−1^, and its energy density is 54.99 Wh kg^−1^.

#### MOFs-Derived Metal Hydroxides

Metal hydroxide has the advantages of low price, wide sources, diverse structures, and high theoretical specific capacitance, which is expected to replace precious metal oxides and become an ideal electrode material. However, metal hydroxide will have serious volume expansion and contraction in the charge and discharge process, worsening its cycle stability. It is not easy to obtain metal hydroxides with excellent specific capacity and cycle stability through simple synthesis methods. The derivatives obtained by high-temperature calcination of MOFs have made some progress in supercapacitors. Still, in the process of high-temperature calcination with MOFs as a template, the structure of MOFs may collapse, or the accumulation of derivatives may occur. When using MOFs as precursor to synthesize metal hydroxide, the metal ions in MOFs can be used as the precursor ions of hydroxide, and the binding force between metal ions and organic ligands is relatively weak. Therefore, MOFs can have an ion exchange reaction with an alkaline solution, and OH^−^ will replace organic ligands to obtain the corresponding hydroxide, effectively avoiding this phenomenon [154, 155]. Zhang et al. [156] synthesized Ni(OH)_2_ by optimizing the hydrolysis temperature of Ni-MOF-74 to transform both internally and externally. The specific capacity of 713.2 C g^−1^ can be achieved at 1 A g^−1^ for the Ni(OH)_2_ electrode prepared under the optimized conditions, which is more than 28% greater than other electrode prepared at other different temperatures. This is because the higher treatment temperature is conducive to the entry of OH^−^ ions into the pores and completes conversion.

**Fig. 13:**
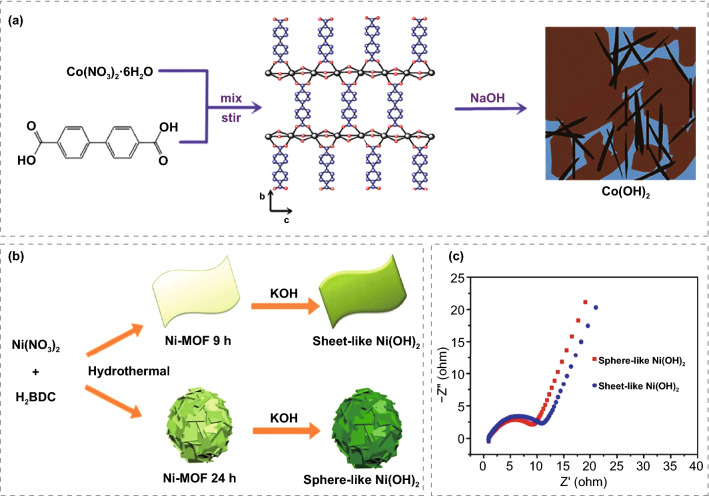
**a** Schematic illustration of the formation process for MOF-derived Co(OH)_2_. Reprinted with permission from Ref. [158]. Copyright 2015 Royal Society of Chemistry. **b** Illustration of the synthetic procedure of sheet-like Ni(OH)_2_ and sphere-like Ni(OH)_2_. **c** The eis of sheet-like and sphere-like Ni(OH)_2_. Reprinted with permission from Ref. [159]. Copyright 2019 Springer Science Business Media

Sun et al. [157] hydrolyzed Ni MOF in KOH aqueous solution through a "conformal transformation" strategy to prepare Ni(OH)_2_ with a layered structure. The electrochemical performance of Ni(OH)_2_ prepared by soaking in 6 M KOH solution for 6 h is the best. The specific capacity of 830.6 C g^−1^ can be achieved at 5 A g^−1^, the specific capacity of 461.2 C g^−1^ can be maintained at 20 A g^−1^. The Ni(OH)_2_ material obtained has the best crystallinity and porosity, which is conducive to electron transport and ion migration in the electrode. The morphology of metal hydroxide is an important factor affecting electrochemical performance. Zhang et al. [158] used Co-MOF as a template and stirred the prepared Co-MOF in NaOH solution through a simple and effective solid-state conversion method. The MOF-derived Co(OH)_2_ can shorten the ion transfer distance, increase the contact area between Co(OH)_2_ and electrolyte, and improve its specific capacitance because of the loose mesoporous structure (Fig. 13a). When the current density is 0.1 A g^−1^, the specific capacitance of 614.6 F g^−1^ can be achieved and exhibits excellent rate performance (the specific capacitance of 430.9 F g^−1^ can be maintained at 10 A g^−1^) and good cycle performance (the specific capacitance retention of 70% can be maintained after 2000 charge/discharge cycles). At the same time, the study also points out that in the synthesizing process of Co(OH)_2_, only cobalt ions and NaOH are consumed. The organic ligands in MOF are only replaced by OH^−^, and the organic ligands will not be destroyed. The recycling of ligands can be accomplished by adjusting the PH of mother liquor, reducing the pollution to the environment, and improving organic ligands' utilization rate. Therefore, this method is environmentally friendly compared with preparing various electrode materials by high-temperature pyrolysis. Tang et al. [159] synthesized flake and spherical Ni(OH)_2_ by precursor conversion method using Ni-MOF as precursor and self-sacrificing template. Ni-MOF as a precursor and self-sacrificial template by a precursor conversion method and subsequently compared the electrochemical properties of flake Ni(OH)_2_ and spherical Ni(OH)_2_ electrodes. The electrochemical results show that spherical Ni(OH)_2_ has better specific capacitance (982 F g^−1^ at 1 A g^−1^), better cycle life, and relatively low resistance; which can be attributed to its porous structure and large specific surface area, providing a large electroactive region and abundant mesoporous for solute diffusion (Fig. 13b, c). It is concluded that spherical Ni(OH)_2_ has a broad application prospect in supercapacitors. Li et al. [160] successfully synthesized honeycomb Ni(OH)_2_, which delivered a high specific capacitance (1865 F g^−1^ at 1 A g^−1^, 59.46% at 10 A g^−1^). The excellent electrochemical performance was attributed to its unique honeycomb structure, which had a very high specific surface area and greatly accelerated the conversion and diffusion of active ions.

## Conclusions

Since discovering MOFs in the 1990s, their sensors, catalysis, and energy storage applications have attracted great attention. MOFs have special pore size distribution, which is beneficial for the diffusion of electrolyte ions and huge specific surface area, increasing the quantity of active sites, making it one of the research hotspots of high-performance electrochemical energy storage materials. MOFs can be directly used as supercapacitor electrodes because the metal ions contained in MOFs have redox activity and can instantly react with electrolyte ions to store energy. However, the poor conductivity of MOFs limits their practical application in the field of energy storage and conversion. In the future, changing the disadvantage of poor structural conduction of MOFs can become a research hotspot within the application of pure MOF materials in energy storage. MOF composites provide a great opportunity for SCs electrode materials with ideal energy and power density. In the synthesis of MOF composites, it is particularly difficult to control the size and position of the composites accurately. Therefore, exploring new synthesis methods to simplify the synthesis steps and improve the properties of composites is the focus of current research. The derivatives prepared with MOFs as a template or precursor have many advantages. For instance, derivatives with different pore properties can be prepared by adjusting the ratio of metal ions to ligands or constructing different structural units to regulate the pore size of MOF materials. In addition, the stability of MOFs is relatively weak, which provides a premise for the synthesis of different derivatives while the process of this method is simple. Concisely, although there are still many problems to be solved before MOF materials are widely used in practical supercapacitors, they still have bright prospects in the area of electrochemical energy storage. Supercapacitors with the high power density and high energy density based on MOF materials will be developed with in-depth research.

## Challenges and Prospects

MOFs have been proved to be a promising electrode material for supercapacitors due to its simple synthesis route and large specific surface area. Compared with traditional materials, MOF materials have obvious advantages. First, the structure of MOF material is adjustable: the size and shape of crystal pores can be adjusted to achieve the purpose of improving performance by selecting organic ligands. Second, the composition of MOF materials is highly diverse. By selecting different organic ligands and metals, MOF materials with different structures can be constructed to achieve different target properties. The third MOF material is porous and can achieve a high specific surface area through proper design which alleviates volume changes and promotes electrolyte penetration. However, there are still some problems to be solved when MOFs is used as electrode in commercial supercapacitors. On the one hand, because the influence of the microstructure and chemical composition of MOFs on the electrochemical storage of constructed supercapacitors is not very clear, how to reveal the structure–activity relationship between the two is of great significance to improve the charge–discharge performance. Although the high specific surface area and porosity of MOFs are conducive to improving the specific capacitance of supercapacitors, the conductivity of MOF materials needs to be improved. The high resistance of MOFs inhibits the improvement of its electrochemical energy storage performance. The design and control of MOFs with various morphologies has become a hot topic in the past few years. Many studies have shown that the low conductivity and structural instability of primitive MOFs can be partially eliminated by designing primitive MOFs into nanostructures with specific geometric morphology, such as 1D nanowires and nanoribbons, 2D nanosheets, and 3D spherical or flower-like structures. These morphological adjustment strategies give new functions and properties to traditional MOF materials. However, the conductivity of most MOFs is extremely low and difficult to solve by adjusting the morphology. Combining MOFs with other excellent energy storage materials to play a synergistic role will be an important development direction for building new electrode materials in the future. At present, various conductive composites of MOFs with carbon or conductive polymers have been prepared and proved to be good electrode active materials in SCs. These composites not only promote the development of new advanced materials, but also broaden the scope of application of MOFs in the field of electrochemistry. At present, the application of MOFs in SC field is only at the initial stage in the laboratory. With the deepening of research on the design, synthesis and function enhancement of MOFs, we believe that green, low-cost and mass excellent MOFs-based energy storage and conversion devices can be commercialized in the near future.
